# Efficacy and safety of elobixibat in patients with chronic constipation—A randomized, multicenter, double-blind, placebo-controlled, parallel-group study from India

**DOI:** 10.1007/s12664-024-01719-7

**Published:** 2025-02-22

**Authors:** Piyush Agarwal, Brajesh Kumar Jha, Jaganmohan Somagoni, Srinivas Shenoy B, Vipul Modh, Sanketh Kumar Chakilam, Vinay Kumar, Mukewar Shrikant Vasantrao, Mukesh Kalla, Anumula Kavitha, Omesh Goyal, Ashima Bhatia

**Affiliations:** 1 Global Clinical Management, Dr. Reddy’s Laboratories Pvt. Ltd, Hyderabad, 500 016 India; 2 GSVM Medical College, Kanpur, 208 002 India; 3 Midas Multispecialty Hospital Pvt. Ltd, Nagpur, 440 010 India; 4 S.R. Kalla Memorial Gastro and General Hospital, Jaipur, 302 006 India; 5 Government General Hospital, Guntur, 522 001 India; 6 Dayanand Medical College and Hospital, Ludhiana, 141 001 India

**Keywords:** Bile acid binding protein, Constipation, Defecation, Elobixibat, Intention to treat analysis

## Abstract

**Background:**

Elobixibat is a locally acting ileal bile acid transporter (IBAT) inhibitor that relieves functional constipation in patients by accelerating colonic transit. In this study, we aimed at determining the efficacy and safety of elobixibat for short-term treatment (two weeks) of chronic constipation in Indian patients.

**Methods:**

The present study was a randomized, double-blind, parallel-group, placebo-controlled, phase III study to evaluate efficacy and safety of elobixibat. The study planned to enroll patients with chronic constipation of at least six months' duration, satisfying Rome IV criteria for functional constipation. Following a run-in of approximately 14 days to confirm eligibility and determine baseline frequency of spontaneous bowel movements (SBMs), eligible patients were randomized 1:1 either to elobixibat or to placebo groups. The change in weekly frequency of spontaneous bowel movements (SBMs) at the end of treatment (week two) over baseline was the primary efficacy endpoint in this trial. Primary efficacy analyses were based on the modified intention-to-treat (mITT) population. This trial is registered at CTRI (Clinical Trial Registry of India).

**Results:**

Between April 2023 and December 2023, 150 patients were randomized into the two-week trial. In mITT population (*n* = 146 [elobixibat = 75 and placebo = 71]), the least square mean (LSM) difference between elobixibat (3.83) and placebo (2.68) was 1.15 (95% CI, 0.31, 1.99) demonstrating a statistically significant improvement (*p* = 0.008) in weekly frequency of SBMs (week two over baseline) with the use of elobixibat. The proportions of patients with a complete spontaneous bowel movement (CSBM) “response” was significantly higher with elobixibat (49.33%) compared to the placebo (26.76%) treatment (difference 22.57% [95% CI, 8.36%, 36.78%] [*p* = 0.005]). The most common adverse event (AE) was abdominal pain (elobixibat = 6 patients [7.89%] vs. placebo = 3 patients [4.05%]).

**Conclusions:**

Elobixibat was well tolerated and improved bowel movement frequency within two weeks of treatment in Indian patient population with chronic constipation.

**Clinical trial registry number:**

CTRI/2022/10/046690

**Graphical Abstract:**

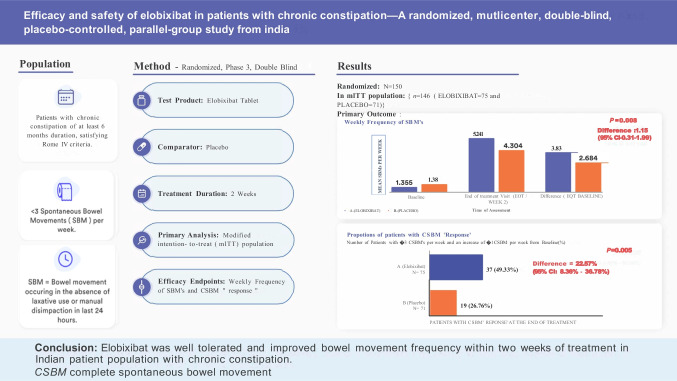

**Supplementary Information:**

The online version contains supplementary material available at 10.1007/s12664-024-01719-7.

## Introduction

Chronic constipation (CC) is the sixth most frequent gastrointestinal complaint, with a prevalence of roughly 16% [1]. The prevalence is higher in non-Caucasians than in Caucasians [2], in women (median female to male ratio of 2–3:1) [3, 4] and in elderly population [2–5].

A population-based study from India found the prevalence of constipation by the Rome II criteria to be 16.8% and self-reported constipation to be 24.8% [6]. Available studies indicate CC is a common health problem in India, with fair evidence obtained from well-designed cohort or case-controlled studies [5]. A comprehensive pan-India multicentric study interviewed 2785 patients with chronic lower gastrointestinal symptoms and 4500 non-complaining subjects. Among those with symptoms, 53% reported self-perceived constipation, while in the non-complaining group, 18% and 23% reported straining during bowel movements and incomplete stool evacuation, respectively [7]. These findings underscore the significance of addressing chronic constipation as a public health concern in India.

The most common symptoms of chronic constipation are hard stools, infrequent bowel movements, straining and a feeling of incomplete evacuation [8]. Symptoms affect individuals of different ages, ethnicities, socio-economic backgrounds and nationalities. These symptoms have a detrimental effect on patients’ quality of life (QOL) and socio-economic status [9].

Contact stimulant laxatives (such as sennoside and sodium picosulfate hydrate), saline laxatives (such as magnesium oxide, lactulose, sorbitol, and polyethylene glycol (PEG)) and medications that change the function of the intestinal epithelium (such as lubiprostone) can be used alone or in combination to treat constipation. Use of these medications is associated with side effects of abdominal pain, diarrhea, nausea, dehydration and electrolyte abnormalities such as hypermagnesemia [10]. Long-term use or abuse of stimulant laxatives is of concern as it has been reported to be associated with increased risk of resistance or habituation and atonic colon [11, 12]. Despite the availability of different treatment options for constipation, the quest for an unmet need for drugs in the treatment of patients with CC continues.

Elobixibat is a novel, minimally absorbed inhibitor of the ileal bile acid transporter (IBAT) that increases the production of bile acids from cholesterol in the liver and blocks the enterohepatic circulation of bile acids (BA) [13]. Upregulation of hepatic BA synthesis promotes maintenance of BA pool size and may also lower serum low-density lipoprotein (LDL) cholesterol concentrations. Increased concentrations of bile acids in the colon enhance transit by dual actions: stimulating fluid and electrolyte secretion and inducing high-amplitude propagated contractions based on the effects of intra-luminal chenodeoxycholate in the human colon [14, 15].

Elobixibat was approved on January 19, 2018, by the Japanese Pharmaceuticals and Medical Devices Agency (PMDA). That clinical development program for elobixibat included a two-week, phase-2b trial [16] and two phase-3 clinical trials [17] (a two-week double-blind placebo-controlled phase-3 trial and an open-label single-arm 52-week long-term phase-3 trial). For the current study, a starting dose of 10 mg elobixibat was selected based on findings of safety and efficacy in the Japanese phase 2b trial [16]. Also, elobixibat showed accelerated colonic transit, looser stool consistency, decreased constipation rating and reduced straining compared with placebo in a phase-2a study [18] over 14 days. Phase-3 clinical trials from Japan indicated that elobixibat was associated with a significantly greater increase in the number of spontaneous bowel movements (SMBs) per week from baseline compared to placebo. Elobixibat also improved bloating severity [17].

The objective of the present trial was to assess the efficacy and safety of once-daily elobixibat for two weeks in Indian patients with chronic constipation.

## Methods

### Study design and participants

This was a prospective, multicenter, randomized, double-blind, parallel-group, placebo-controlled, phase III study to evaluate the efficacy and safety of elobixibat tablet in patients with chronic constipation.

The study planned to enroll eligible male and female patients aged 18 to 65 years (both inclusive), with chronic constipation of at least six months' duration. Patients were diagnosed on the basis of standard symptom-based criteria of fewer than three SBMs per week (defined as bowel movements occurring spontaneously and independently of administration of rescue medication for at least 24 hours), with at least one of the following symptoms during 25% or more of bowel movements: straining at defecation, lumpy or hard stools and sensation of incomplete evacuation. These are included in Rome IV criteria for functional constipation [19].

Patients experiencing constipation needed to have documented results from a colonoscopy or sigmoidoscopy conducted within two years leading up to their screening visit. This requirement was put in place to rule out any organic causes for constipation.

Patients having any cardiovascular, liver, renal or psychiatric disorder or any known adverse drug reaction (ADR) to study drug and excipients were excluded. Pregnant and breast-feeding women were also excluded from our study. The following conditions disqualified patients from participation: prior intestine or rectum surgery (except for a simple appendectomy); prohibited medications, organic disorders of the intestine such as mechanical obstruction; ischemic colitis, inflammatory bowel disease, colorectal cancers and pre-malignant colonic disease (e.g. familial adenomatous polyposis or hereditary non-polyposis colorectal cancer) or other forms of familial colorectal cancer. The requirement for screening colonoscopy was waived for individual patients under 45 years of age, provided they had not experienced alarm symptoms such as weight loss, rectal bleeding or anemia in the past six months.

The study consisted sequentially of a screening period of 14 days, a “pre-treatment” (run-in) period of 14 days followed by the randomized treatment period. The treatment period started with randomization visit (Day 0) and continued for two weeks. Primary efficacy and end of study assessments were performed on Day 14. Patients reporting more than 3 SBM per week on average during pre-treatment period were excluded from the study.

### Intervention and randomization

Eligible patients, recruited from 16 clinical sites in India, were randomized using interactive web response systems (IWRS) in 1:1 ratio (either to elobixibat or to placebo treatment groups). Randomized patients took the assigned study drug once per day before breakfast and were monitored in an outpatient setting. Per assignment, each randomized patient had to take two tablets of the investigational product (elobixibat 5 mg or placebo) once a day orally. In the second week, down titration of dose to one tablet or up titration to three tablets was permitted in individual patients based on symptoms. Placebo tablets matching test products in appearance were used.

### Allocation concealment

Study drugs (elobixibat or placebo with identical appearance) were supplied in over labeled blister packs and each blister was labeled with a unique kit number. Group assignment was concealed from patients, investigators, sponsors and data analysts.

### Implementation of blinding

The patients and investigator (and other personnel involved in the study) were unaware of the study drug(s) administered to patients. The sponsor was also blinded during the study. The placebo tablets and its packaging and labeling were identical in appearance to that of test products.

### Study objectives and outcomes

The primary objective of the study was to evaluate the efficacy of elobixibat. Primary efficacy variable was weekly frequency of SBMs. The change in weekly frequency of SBMs at the end of treatment (week two) over baseline was the primary efficacy endpoint in this trial. Each patient’s weekly SBM frequency was calculated using the following formula: weekly SBM frequency = 7 × (total number of SBMs during the treatment period)/(number of days of treatment period considered). Use of rescue medication (5 mg bisacodyl tablets or 10 mg bisacodyl suppositories) was permitted if the patient had no bowel movement for 72 hours or longer and this was recorded in the diary for consideration during the efficacy analysis. Bowel movements that followed within 24 hours of rescue medication usage were excluded from computations of SBM frequency.

Secondary efficacy endpoints included proportion of patients with complete spontaneous bowel movement (CSBM) “response” (CSBM responder was defined as a patient with ≥ 3 CSBMs per week and an increase of ≥ 1 CSBM per week from baseline), proportion of patients with a SBM within 24 hours after the first dose of study drug, median time to first SBM, changes in stool consistency score (scored using the seven-point Bristol Stool Form Scale [BSFS]) [20], degree of straining of SBMs and abdominal bloating score (using the five-point ordinal scale) over baseline.

### Determination of sample size

Assuming a significance level of 2.5% (two sided), power of 95%, test group mean response 6, placebo group mean response 2 and a pooled standard deviation (SD) of 6 [17], it was estimated that 60 completed patients per treatment group (a total of 120 patients in the study) would be required to prove superiority of elobixibat. Assuming ~ 20% drop-out rates, 150 patients were planned to be randomized in this study with 1:1 ratio (75 patients per arm) to elobixibat and placebo.

### Statistical analysis

All descriptive and inferential statistical analyses were performed using SAS® version 9.4 in a secure and validated environment, unless otherwise noted. Statistical significance was concluded when the *p*-value was less than 0.05. Continuous data was summarized in terms of the mean, SD, median, 95% CI, minimum, maximum and number of observations, unless otherwise stated. Categorical data were summarized in terms of the number of patients providing data at the relevant time point (*n*), frequency counts and percentages.

All randomized patients who met all inclusion/exclusion criteria administered at least one dose of assigned investigational product and had at least one post-baseline evaluation of the primary estimate were included in the mITT population.

Mean difference between treatments was analyzed using analysis of covariance (ANCOVA) with change from baseline to two weeks as the dependent variable and treatment group and baseline no. of SBMs, center, age and sex as covariates. The two-sided 95% CI and associated *p*-value were computed for the least square mean (LSM) difference between the elobixibat and placebo drug.

The proportion difference was tested using Chi-square test to assess whether changes in the efficacy parameters are significant in the groups in comparison (elobixibat vs. placebo) of interest. Non-parametric tests were used where appropriate for evaluation of association between different variables. No multiplicity adjustments were made during analysis of the secondary endpoints.

Safety population included all randomized patients who received at least one dose of the investigational drug were included in the safety population. All safety analyses were based upon the safety population.

### Data collection and management

During the study, patients were asked to fill in paper diaries daily in the two-week treatment period. The following assessments were to be recorded by patients in the provided paper diaries: time of each bowel movement, stool consistency (scored with the seven-point Bristol Stool Form Scale [BSFS]), sensation of complete bowel emptying and use of rescue medication. Severity of constipation (using the five-point adjectival scale: none, mild, moderate, severe and very severe) was to be recorded once weekly. Patients continued to provide their daily assessments, their weekly assessments and their use of rescue medicine and any other laxatives, suppositories or enemas up to two weeks or end of treatment (EOT).

Electronic case record forms (CRFs) were used to collect information required for data analysis. When the database was declared to be complete and accurate, the database was locked and unblinded.

### Ethical considerations

The study was conducted in compliance with the ethical principles that originate in the Declaration of Helsinki and the International Conference on Harmonization (ICH) guidelines for good clinical practice (GCP). The study was approved by institutional ethics committee of each participating centers. Written informed consent was obtained from each patient prior to screening on the approved informed consent form (ICF). Patients received a defined conveyance allowance in the study but were not compensated in other manner. This trial is registered at CTRI (Clinical Trial Registry of India) with the Trial Registration number CTRI/2022/10/046690 (The final protocol for the study is provided as a Supplementary file-appendix 1).

## Results

### Study population characteristics

**Fig. 1:**
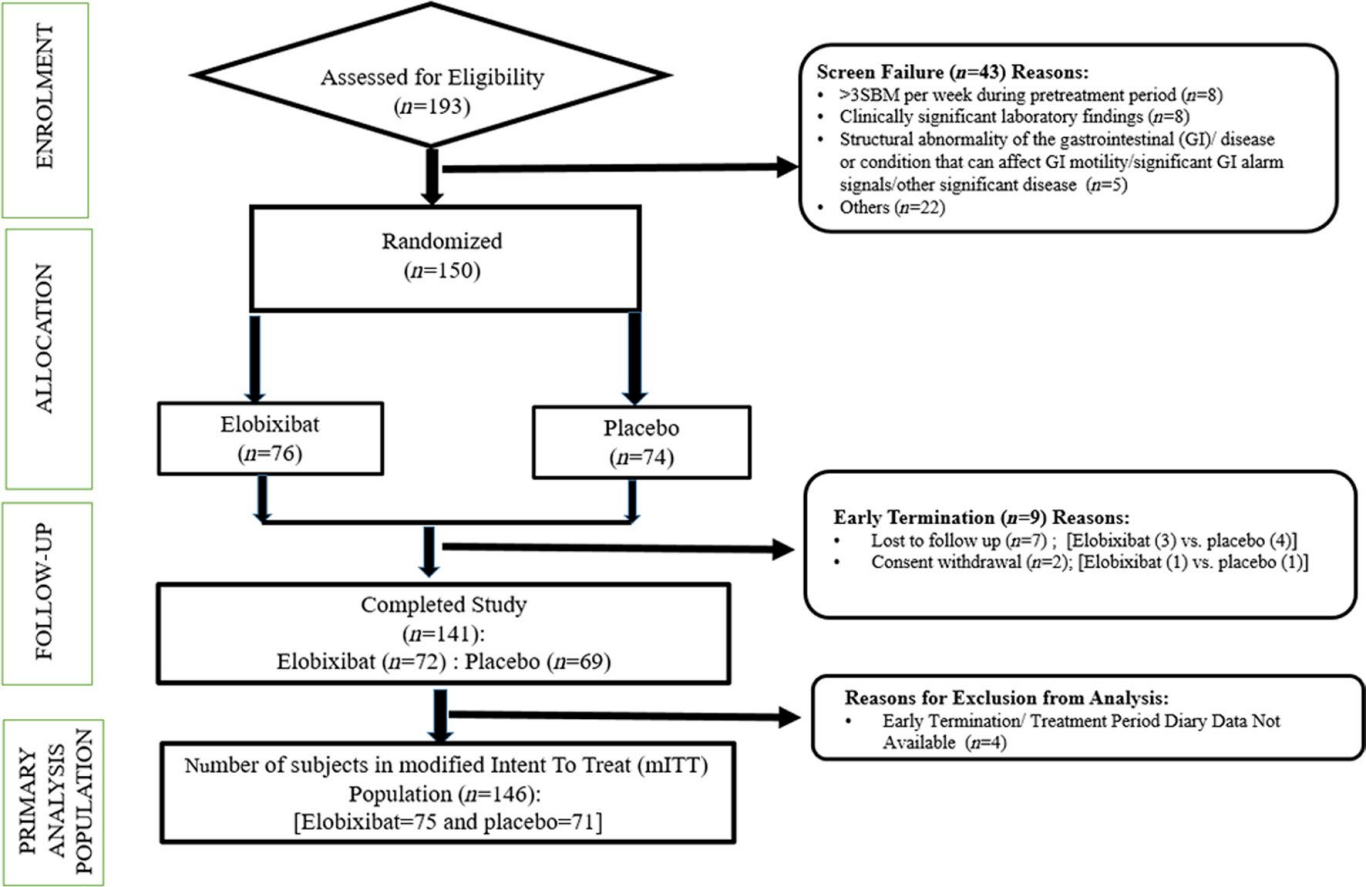
Consolidated Standards of Reporting Trials (CONSORT) diagram displaying the flow of participants through the study

Between April 2023 and December 2023, total 193 patients were screened at 16 sites across India, of which 150 patients were randomized and 43 patients were screen failures (Fig. 1). All 150 patients (who received at least one dose of study drug) were included in the safety analysis population and 146 patients (97.33%) were in modified Intent-to-treat (mITT) population (elobixibat = 75 and placebo = 71).

Of the 150 patients who received at least one dose of study drug, 100 (66.67%) were males and 50 (33.33%) were females. The median age of enrolled patients was 39.5 years (ranging from 18 to 64 years). The mean (SD) baseline body mass index (BMI) was 24.3 (4.51) (Table 1). All patients were Asian (Indian). Demographic characteristics were comparable between the two treatment groups.

**Table 1:** Summary of baseline demography

Demographic variable	Statistic	Elobixibat (*n* = 76)	Placebo (*n* = 74)	Total (*n* = 150)	*p*-value
Age (year)	Mean (SD)	42.1 (12.80)	38.2 (12.92)	40.2 (12.97)	0.0633
	Median (min–max)	43.5 (18.0–64.0)	36.0 (18.0–64.0)	39.5 (18.0–64.0)	
Gender	Female	28 (36.84%)	22 (29.73%)	50 (33.33%)	0.3556
	Male	48 (63.16%)	52 (70.27%)	100 (66.67%)	
BMI (kg/m^2^)	Mean (SD)	24.5 (4.64)	24.0 (4.40)	24.3 (4.51)	0.5671
	Median (min–max)	24.3 (12.7–36.4)	23.6 (15.0–33.3)	23.9 (12.7–36.4)	

*SD* standard deviation, *BMI* body mass index, *cm* centimeter, *kg* kilogram, *m* meter, *min–max* minimum–maximum

Demographic characteristics were compared between treatment groups using two sample *t*-test for *p*-value calculation. *p*-value < 0.05 was considered significant

### Efficacy results

In primary analysis (*n* = 146 [elobixibat = 75 and placebo = 71] [mITT population]), the LS mean difference between elobixibat (3.83) and placebo (2.68) was 1.15 (95% CI, 0.31, 1.99), demonstrating a statistically significant improvement (*p* = 0.008) in weekly frequency of SBMs with the use of elobixibat. Similarly, elobixibat exhibited a statistically significant difference in proportion of patients with CSBM “response”; difference in proportions between elobixibat (49.33%) and placebo (26.76%) was 22.57% (95% CI, 8.36, 36.78) (*p* = 0.005). Within 24 hours of initiation of study treatment, 51% of patients in the elobixibat group and 37% in the placebo group reported an SBM; however, the difference (14.05% [95% CI, − 0.69 to 28.78]) did not achieve statistical significance (*p* = 0.087) (Table 2). Additionally, median time (HH:MM) to first SBM was shorter in the elobixibat group (11:26; [IQR, 24.20; Q1, 2:00 to Q3, 26:20]) compared to placebo (22:30; [IQR, 40:32; Q1, 4:25 to Q3, 44:57]) showing clinically meaningful onset of action with the use of elobixibat. The difference, however, was not statistically significant (*p* = 0.143) (Table 3). Results on other endpoints were statistically non-significant and equivocal for elobixibat and placebo in this trial (Table 2).

**Table 2:** Summary of efficacy

Continuous efficacy variables	Elobixibat (*n* = 75)				Placebo (*n* = 71)				LSM difference [90% CI]	***p*-**value^a^
	LSM at baseline	LSM at week 2		LSM difference^**c**^	LSM at baseline	LSM at week 2		LSM difference^c^		
Weekly frequency of SBMs	1.3546	5.2408		3.8297	1.3803	4.3044		2.6838	1.15 [0.31, 1.99]	0.008
Stool consistency using BSFS	2.7066	3.3996		0.6930	2.8425	3.3941		0.5515	0.14 [− 0.13, 0.41]	0.307
Weekly degree of straining at defecation	3.2536	2.5948		0.658	3.3752	2.7545		− 0.6207	− 0.04 [− 0.27, 0.19]	0.744
Abdominal bloating score	3.1174	2.4763		− 0.6411	3.2221	2.5921		− 0.6300	− 0.01 [− 0.28, 0.25]	0.934
Abdominal discomfort score	3.0481	2.3355		− 0.7126	3.0714	2.3894		− 0.6821	− 0.03 [− 0.31, 0.25]	0.831
Categorical efficacy variable	Elobixibat (*n* = 75)				Placebo (*n* = 71)				Percentage difference [90% CI]	***p*-**value^b^
	Frequency		Percentage (%)		Frequency		Percentage (%)			
Proportion of patients with CSBM response	37		49.33%		19		26.76%		22.57% [8.36, 36.78]	0.005
Proportion of patients with SBM ≤ 24 h after first dose	38		50.67%		26		36.62%		14.05% [− 0.69, 28.78]	0.087

*CI* confidence interval, *BSFS* Bristol stool form scale, *CSBM* complete spontaneous bowel movement, *LSM* least square mean, *SBM* spontaneous bowel movement

^a^LSM change from baseline was computed using analysis of covariance (ANCOVA) models and two sample *t*-test was utilized for *p*-value and two-sided 95% CI

^b^Study treatment-between group comparison was analyzed using Chi-square test

^c^The LSM difference of the change at Week 2 over baseline

**Table 3:** Median time to first spontaneous bowel movement

Treatment group	Mediantime (HH:MM)	Interquartile range time (HH:MM)	Q1 time (HH:MM)	Q3 time (HH:MM)	*p***-**value
Elobixibat (*n* = 75)	11:26	24:20	2:00	26:20	0.143
Placebo (*n* = 71)	22:30	40:32	4:25	44:57	

*SD* standard deviation, *HH:MM* hours:minutes

- Q1 is the 25th percentile of the data

- Q3 is the 75th percentile of the data

Median time to first SBM between group comparison was calculated using the Wilcoxon signed-rank test

### Safety results

Of the 150 patients included in the safety analyses, 23 patients (15.33%) reported at least one adverse event (AE). The distribution of patients with AEs between treatment groups elobixibat and placebo was fairly even, at 12 (15.79%) and 11 (14.86%), respectively. AEs were categorized as mild (*n* = 57 [95.00%]; elobixibat = 36 [60.00%] vs. placebo = 21[35.00%]) and moderate (*n* = 3 [5%]); elobixibat = 3 (5.00%) vs. placebo = 0] in severity. The most common AEs were abdominal pain (elobixibat = 6 patients [7.89%] vs. placebo = 3 patients [4.05%]), abdominal distention (elobixibat = 3 patients [3.95%] vs. placebo = 3 patients [4.05%]) and dry mouth (elobixibat = 2 patients [2.63%] vs. placebo = 1 [1.35%] patients). No deaths or serious adverse events (SAEs) occurred in the study (Table 4). No clinically significant abnormalities were observed in vital signs, clinical laboratory parameters and physical examination data.

**Table 4:** Summary of adverse events (AEs)-safety population

Description	Elobixibat *n* (%)	Placebo *n* (%)	Total *n* (%)
Patients randomized	76	74	150
Patients with at least one AE	12 (15.79%)	11 (14.86%)	23 (15.33%)
Patients who discontinued study drug due to AEs	0 (0.00%)	0 (0.00%)	0 (0.00%)
No. of AEs reported	39 (65.00%)	21 (35.00%)	60 (100.0%)
Mild	36 (60.00%)	21 (35.00%)	57 (95.00%)
Moderate	3 ( 5.00%)	0 ( 0.00%)	3 (5.00%)
Common adverse events	Patients *n* (%)	Patients *n* (%)	Patients *n* (%)
Abdominal pain	6 (7.89%)	3 (4.05%)	9 (6.0%)
Abdominal distension	3 (3.95%)	3 (4.05%)	6 (4%)
Dyspepsia	2 (2.63%)	1 (1.35%)	3 (2%)

## Discussion

The study demonstrated a statistically significant improvement in the weekly frequency of spontaneous bowel movements with a two-week treatment with elobixibat self-administered once daily, in comparison to placebo. A statistically significant separation between the treatment groups was also observed for the secondary endpoint on complete spontaneous bowel movement responder analysis in the two-week treatment period. A sizeable and clinically meaningful difference in proportion of patients with SBM within 24 hours of first administration of study treatment and an earlier median time to first SBM was observed with elobixibat treatment, although the differences observed in the current study were not statistically significant. Because many patients take medications for constipation intermittently, the shorter median time to induce a bowel movement with elobixibat is clinically beneficial. This could be attributed to the known mechanism of action of IBAT inhibitors, accelerating colonic transit through dual biological effects of increased colonic bile acid concentrations on intestinal fluid secretion and motility.

These findings are consistent with previous phase 2b, two-week trial and randomized phase III trials from Japan elucidating the effects of elobixibat on SBM and CSBM response [16, 17].

On the other secondary endpoints, the elobixibat group showed comparable improvements over baseline at two weeks to the placebo group with no significant difference.

This is the first randomized, double-blind and placebo-controlled trial demonstrating efficacy and safety of elobixibat in Indian patients with chronic functional constipation. Patients meeting definition of chronic functional constipation as per ROME IV criteria were recruited for the study from centers across India and therefore, the observed outcomes are generalizable to Indian patients with chronic functional constipation.

The potential limitation of the study may include the apparent lack of efficacy on the subjective scoring endpoints which could possibly be due to poor comprehension of the scoring system by patients, diary compliance issues or a recall bias.

Pertinently, a significant proportion of patients underwent intensive treatment with osmotically active laxatives as part of bowel preparation for colonoscopy during the screening period, the effects of which may have persisted into the pre-treatment and randomized treatment periods, which could likely explain the high placebo response and lack of separation on some of the secondary endpoints. Notwithstanding this “carryover” effect, elobixibat treatment has shown a positive difference over placebo on more objective endpoints based on SBM and CSBM frequency at week one, which culminated into a statistically significant difference at the primary assessment at week two. Waning of the effects of bowel preparation over time in the placebo group has likely been added to its separation from elobixibat at the primary assessment timepoint.

Overall, elobixibat was found to be generally safe and well-tolerated over two weeks of treatment. There were no SAEs or deaths reported in the study.

In conclusion, this study demonstrated that elobixibat, an ileal bile acid transporter inhibitor, was effective in short-term treatment of chronic constipation in Indian patients and was well-tolerated. Good tolerability, earlier onset of action, short treatment durations and convenient once-daily dosing are tangible benefits with the IBAT inhibitor class of drugs, augmenting the available therapeutic armamentarium for poorly understood and often difficult to treat chronic functional constipation.

## Supplementary Information

Below is the link to the electronic supplementary material.

Supplementary file1 (PDF 1322 KB)

Supplementary file2 (DOC 220 KB)

Supplementary file3 (DOCX 16 KB)

## Data Availability

The data that support the findings of this study is available from the corresponding author, upon reasonable request.

## Declarations

### Disclaimer

The authors are solely responsible for the data and the contents of the paper. In no way, the Honorary Editor-in-Chief, Editorial Board Members, the Indian Society of Gastroenterology or the printer/publishers are responsible for the results/findings and content of this article
