# Supplementary material for: Efficacy and safety of elobixibat in patients with chronic constipation—A randomized, multicenter, double-blind, placebo-controlled, parallel-group study from India
Source: Indian J Gastroenterol. 2025 Feb 22;44(3):336–44. doi: 10.1007/s12664-024-01719-7 (PMC12141165; doi:10.1007/s12664-024-01719-7)
Supplement: Supplementary file 1 — Supplementary file1 (PDF 1322 KB) [file 12664_2024_1719_MOESM1_ESM.pdf]

TITLE

A RANDOMIZED, MULTICENTRE, DOUBLE BLIND, PLACEBO CONTROLLED, PARALLEL-GROUP STUDY TO EVALUATE THE EFFICACY AND SAFETY OF ELOBIXIBAT ONCE DAILY OF DR. REDDY'S LABORATORIES LIMITED IN PATIENTS WITH CHRONIC CONSTIPATION

**Sponsor**	Dr. Reddy's Laboratories Ltd.<br>Integrated Product Development,<br>Innovation plaza,<br>Survey No: 42, 45 and 46,<br>Bachupally village, Bachupally Mandal<br>Medchal Malkajgiri District<br>500 090, Telangana, India.
**Protocol Title.**	A randomized, multicentre, double blind, placebo controlled, parallel-group study to evaluate the efficacy and safety of elobixibat once daily of Dr. Reddy's laboratories limited in patients with chronic constipation
**Protocol ID.**	DRL-IND-NDA03-ELO/2022
**Study Drug Name**	Elobixibat
**Development Phase**	Phase III
**Version and Date of Protocol**	Version 2.0, 17-OCT-2023
**Preceding version and date**	Version 1.0; 11-MAR-2022
**Protocol Amendment(s) if any**	NA
**Amendment no & date**	NA

The study will be conducted according to the protocol and in compliance with Good Clinical Practice (GCP), and with other applicable regulatory requirements.

DocuSign Envelope ID: DCBE1147-372F-4B2F-B3C1-63958E711F1B

This document is the property of Dr. Reddy's Laboratories Limited and is confidential. Therefore, it may not be photocopied, either in part or in full, or disclosed to any individual not directly associated either with the trial, or with the concerned ethics committee, or with the concerned regulatory authority

SPONSOR:

Dr. Reddy's Laboratories Ltd. Integrated Product Development,
Innovation plaza, Survey No: 42, 45 and 46,
Bachupally village, Bachupally Mandal
Medchal Malkajgiri District 500 090, Telangana, India.

PROTOCOL VERSION HISTORY

Version No	Date	Change in protocol	Reason
Version 1.0	11-MAR-2022	Yes	For clarification on colonoscopy requirement
Version 2.0	17-OCT-2023	No	

Summary of Changes from Version 1.0 dated 11-MAR-2022 to Version 2.0 dated 17-OCT-2023

Sl. No	Section(s) changed in the protocol version 1.0 dated 11-MAR-2022	Initial statement	Proposed change	Reason for change	Anticipated impact, if any
1	Exclusion criteria no.2	Subject of any age with evidence of clinically significant weight loss, anemia, or rectal bleeding and without documentation of the results of either a flexible sigmoidoscopy or colonoscopy performed within 2 years period prior to screening.	Subject of any age with evidence of clinically significant weight loss, anemia, or rectal bleeding and without documentation of the results of either a flexible sigmoidoscopy or colonoscopy performed within 2 years period prior to screening.<br>**Note:** Colonoscopy can be avoided for patients age less than 45 years and those who do not have alarming symptoms of weight loss, rectal bleeding or anemia in past 6 months based on Investigator discretion/ Medical Monitor opinion.	Clarification on colonoscopy requirement	None
2	Section 4.1(Overview of the Study Design-Treatment period).	Patient will be advised to take study drug once daily in the morning at least 30 minutes before breakfast from Day 1.	Patient will be advised to take study drug once daily in the **morning before breakfast** from Day 1.	Typographical error	None
3	Appendix 2 (Study personnel): Sponsor contact	Dr. Shradhanand Singh<br>Lead –Clinical Operation and Strategy<br>Dr. Reddy's Laboratories Ltd.<br>Email ID:shradhanandsingh@drreddys.com	Dr. Piyush Agarwal<br>Head Clinical Development & Clinical Strategy<br>Dr. Reddy's Laboratories Ltd.<br>Email ID: piyushagarwal@drreddys.com	Administrative change	None

DocuSign Envelope ID: DCBE1147-372F-4B2F-B3C1-63958E711F1B

SIGNATURE PAGE

Declaration of Sponsor Representative

TITLE: A RANDOMIZED, MULTICENTRE, DOUBLE BLIND, PLACEBO CONTROLLED, PARALLEL-GROUP STUDY TO EVALUATE THE EFFICACY AND SAFETY OF ELOBIXIBAT ONCE DAILY OF DR. REDDY'S LABORATORIES LIMITED IN PATIENTS WITH CHRONIC CONSTIPATION.

This study protocol was subjected to critical review. The information it contains is consistent with current knowledge of the risks and benefits of the investigational product, as well as with the applicable guidelines on Good Clinical Practice.

DocuSigned by:
Piyush Agarwal
7808B664909745C...

17-Oct-2023 | 2:39 PM IST

Signatory's name & designation **Date**

Dr. Piyush Agarwal

Head Clinical Development & Clinical Strategy,

Dr. Reddy's Laboratories Ltd.
Integrated Product Development Organization,

Innovation Plaza, Survey no.'s.: 42, 45, and 46,

Bachupally village, Bachupally Mandal,

Medchal-Malkajgiri district,

Telangana-500 090, Hyderabad, India.

Tel: +91 9619097755

E-mail: piyushagarwal@drreddys.com

DocuSign Envelope ID: DCBE1147-372F-4B2F-B3C1-63958E711F1B

DECLARATION BY THE INVESTIGATOR

TITLE: A RANDOMIZED, MULTICENTRE, DOUBLE BLIND, PLACEBO CONTROLLED, PARALLEL-GROUP STUDY TO EVALUATE THE EFFICACY AND SAFETY OF ELOBIXIBAT ONCE DAILY OF DR. REDDY'S LABORATORIES LIMITED IN PATIENTS WITH CHRONIC CONSTIPATION.

All required documents for this study have been provided to me and I confirm that these have not been previously published and will be kept in the strictest confidence. This includes this study protocol and the medical, clinical and scientific data therein.

The study will not be commenced without the prior written approval of a properly constituted Ethics committee (EC). No changes will be made to the study protocol without the prior written approval of the Sponsor and the EC, except where necessary to eliminate an immediate hazard to the subjects.

I have read and understood and agree to abide by all the conditions and instructions contained in this protocol.

Principal Investigator of the study center:

______________________________ ______________________

Signature Date

____

Name (block letters)

____

Title (block letters)

____

Institution (block letters)

DocuSign Envelope ID: DCBE1147-372F-4B2F-B3C1-63958E711F1B

PROTOCOL SYNOPSIS

Title of study	A randomized, multicentre, double blind, placebo controlled, parallel-group study to evaluate the efficacy and safety of Elobixibat once daily of Dr. Reddy's laboratories limited in patients with chronic constipation.
Protocol ID	DRL-IND-NDA03-ELO/2022
Sponsor	Dr. Reddy's Laboratories Limited
Phase of Study	Phase III
Study rationale in brief	Constipation is defined by bowel disturbances (i.e., reduced frequency of bowel habits, hard stools, excessive straining to defecate, a sense of anorectal blockage, anal digitation, and a sense of incomplete evacuation after defecation).<br><br>Chronic constipation (CC) is common among the elderly population. Many challenges are associated with the diagnosis and management of patients with CC. Some of these challenges arise from the current incomplete understanding of what causes constipation and from the difficulties in diagnosing and classifying the heterogeneous group of patients with CC.<br><br>Constipation is labelled as 'Chronic idiopathic constipation (CIC)' when the cause cannot be identified. Global prevalence of CIC is 20%. Population based study from India in 505 people found the prevalence of constipation by the Rome II criteria to be 16.8% and self-reported constipation to be 24.8% in the last 1 year.

DocuSign Envelope ID: DCBE1147-372F-4B2F-B3C1-63958E711F1B

	Despite the availability of different treatment options for constipation, an unmet need for drugs in the treatment of patients with CC remains. A subset of patients with CC have reduced fecal (and by inference, intra-colonic) bile acids (BA).<br><br>Elobixibat represents a new class of treatment for CC with a unique MOA. It is a locally-acting ileal bile acid transporter (IBAT) inhibitor, a protein that regulates reabsorption of BA. This increases the delivery of BA delivery to the colon thereby enhancing natural defecation.<br><br>Inhibition of ileal BA absorption also up regulates hepatic synthesis of BA from cholesterol, which enters into hepatocytes as LDL by binding to LDL receptors on the hepatocyte cell surface. Up regulation of hepatic BA synthesis promotes maintenance of BA pool size and may also lower serum LDL cholesterol concentrations.<br><br>Elobixibat showed accelerated colonic transit, looser stool consistency, decreased constipation rating, and reduced straining compared with placebo in a Phase IIa study over 14 days. Elobixibat was associated with a significantly greater increase in the number of SBMs (spontaneous bowel movements) per week from baseline to placebo. Elobixibat also improves bloating severity.<br><br>This study is planned to evaluate the efficacy, and safety of Elobixibat tablet (10 mg oral once daily administered as two tablets of 5 mg) in patients with chronic constipation once daily for one week and then

	dose titration (up or down) will be done according to the patient's response.
Investigational Medicinal Product	Elobixibat 5 mg tablet
Comparative<br>Medicinal Product	Placebo matching Elobixibat 5 mg tablet
Study Type	Interventional
Study Design	Prospective, multicenter, randomized, double blind, parallel group, placebo controlled, phase III superiority study
Primary Objective	To evaluate the efficacy of Elobixibat tablet in patients with chronic constipation.
Secondary Objective	To evaluate the safety and tolerability of Elobixibat tablet in patients with chronic constipation.
Sample size calculation	A total of 150 Patients with chronic constipation.<br>Assuming a significance level of 2.5% and a power of 95%, we require 60 completed subjects per treatment group (a total of 120 subjects in study) to prove superiority of Elobixibat over Placebo.<br>Assuming ~20% drop-out rates, 150 subjects will be randomized in this study with 1:1 ratio (75 subjects per each arm) to Elobixibat 5mg vs Placebo.
Subjects/clinical condition/therapeutic area	Male and non-pregnant female subjects (18-65 years of age) with clinical diagnosis of chronic constipation will be included in the trial.
Key Inclusion Criteria	Subjects will be enrolled in the study if they meet all the following criteria.<br>1. Male or female patients of 18-65 years age

	2. Ambulatory patients with chronic constipation (fulfilling Rome IV criteria) reporting <3 Spontaneous Bowel Movements (SBM) per week and reports one or more of the following symptoms for the last 3 months with symptom onset at least 6 months before the Screening Visit or before starting chronic therapy with any laxative:<br>• Straining during more than 25% of defecations<br>• Lumpy or hard stools more than 25% of defecations<br>• Sensation of incomplete evacuation more than 25% of defecations<br>3. Patients with less than 3 SBM per week during pretreatment period.<br>4. Willing to comply with protocol requirements and sign a statement of informed consent<br>5. Willing to discontinue any laxatives used before the Pretreatment period in favor of the protocol-defined Rescue Medicine (bisacodyl tablets or suppositories)<br>6. Agreed to refrain from making any new, major life-style changes that may have affected CIC symptoms (e.g., starting a new diet or changing his or her exercise pattern) from the time of signature of the ICF to the last trial visit.<br>7. Female subjects of:<br>Child-bearing potential should have negative serum pregnancy test at screening and agree to use adequate birth control during the entire study period (acceptable methods include intrauterine device, barrier or abstinence).<br>OR

	Is surgically sterile (bilateral tubal ligation, bilateral oophorectomy, or hysterectomy has been performed on the study subject).<br>OR<br>Postmenopausal for at least 1 year
Key Exclusion Criteria	1. Pregnant, breast feeding, or planning a pregnancy<br>2. Subject of any age with evidence of clinically significant weight loss, anemia, or rectal bleeding and without documentation of the results of either a flexible sigmoidoscopy or colonoscopy performed within 2 years period prior to screening.<br>**Note:** Colonoscopy can be avoided for patients age less than 45 years and those who do not have alarming symptoms of weight loss, rectal bleeding or anemia in past 6 months based on Investigator discretion/ Medical Monitor opinion.<br>3. Reports loose (mushy) or watery stools in the absence of any laxative intake in the form of a tablet, a suppository or an enema, or prohibited medicine for >25% of bowel movements.<br>*4.* Reported a Bristol Stool Form Scale score of 6 (loose, mushy stools) for > 1 SBM or a Bristol Stool Form Scale score of 7 (watery stools) with any SBM over the 2 weeks pretreatment period before the Randomization *Visit [Refer to appendix 3 for BSFS scale]*.<br>5. Received a diagnosis of inflammatory bowel disease (IBD)

6. Meet the Rome IV criteria for Irritable Bowel Syndrome or the Rome IV criteria for Opioid-Induced Constipation as described below:

Rome Criteria for IBS and OIC

**IRRITABLE BOWEL SYNDROME**<br>*Diagnostic criteria**	**OPIOID-INDUCED CONSTIPATION**<br>*Diagnostic criteria*
Recurrent abdominal pain on average at least 1 day/week in the last 3 months, associated with two or more of the following criteria:	1. New, or worsening, symptoms of constipation when initiating, changing, or increasing opioid therapy, that must include two or more of the following:
Related to defecation	• Straining during more than ¼ (25%) of defecations
Associated with a change in frequency of stool	• Lumpy or hard stools (Bristol Stool Form Scale 1-2) more than ¼ (25%) of defecations
Associated with a change in form (appearance) of stool	2. Sensation of incomplete evacuation more than ¼ (25%) of defecations<br>3. Sensation of anorectal obstruction/blockage more than ¼ (25%) of defecations
* Criteria fulfilled for the last 3 months with symptom onset at least 6 months prior to diagnosis	4. Manual maneuvers to facilitate more than ¼ (25%) of defecations (e.g., digital evacuation, support of the pelvic floor)
	5. Fewer than three SBM per week<br>6. Loose stools are rarely present without the use of laxatives.

IBS: Irritable Bowel Syndrome, OIC: Opioid Induced Constipation, SBM: Spontaneous Bowel Movement

7. Has a history of structural abnormality of the Gastrointestinal (GI) tract or a disease or condition that can affect GI motility.

8. Has a history of diverticulitis, chronic pancreatitis, active peptic ulcer disease (PUD) not adequately treated, ischaemic colitis, inflammatory bowel disease, laxative abuse, faecal impaction that required

	hospitalization or emergency treatment, pseudo-obstruction, mega colon, mega rectum, bowel obstruction, descending perineum syndrome, ovarian cysts, endometriosis, solitary rectal ulcer syndrome, systemic sclerosis, pre-malignant colonic disease (e.g., familial adenomatous polyposis or hereditary non-polyposis colorectal cancer) or other forms of familial colorectal cancer.<br>9. Diagnosis or family history of familial adenomatous polyposis, hereditary nonpolyposis colorectal cancer, or any other form of familial colorectal cancer<br>10. Current active peptic ulcer disease (i.e., disease that was not adequately treated or stable with therapy)<br>11. Has unexplained and clinically significant GI alarm signals (e.g., lower GI bleeding or heme-positive stool in the absence of known internal or external haemorrhoids, iron-deficiency anaemia, unexplained weight loss) or systemic signs of infection or colitis<br>12. Has a potential central nervous system (CNS) cause of constipation (e.g., Parkinson's disease, spinal cord injury, multiple sclerosis)<br>13. Has intestinal/rectal prolapse or other known pelvic floor dysfunction<br>14. Commonly uses digital maneuvers (perianal pressure or digital disimpaction) or vaginal splinting to facilitate the passage of a bowel movement<br>15. Has a history of diabetic neuropathy<br>16. Bariatric surgery for treatment of obesity, or surgery to remove a segment of the GI tract at any time before the Screening Visit, surgery of the abdomen, pelvis, or

	retroperitoneal structures during the 6 months before the Screening Visit, an appendectomy or cholecystectomy during the 60 days before the Screening Visit, other major surgery during the 30 days before the Screening Visit.<br>17. Has a history of cancer with last date of proven disease activity/presence of malignancy within 5 years, except for adequately treated basal cell carcinoma of the skin, cervical dysplasia, or carcinoma in situ of the skin or the cervix<br>18. Known human immunodeficiency virus (HIV) or Hepatitis B/C (HBV/HCV) infection<br>19. Has a history of hospitalization for any psychiatric disorder, or any suicide attempt in the 2 years prior to Screening<br>20. History of alcohol or drug abuse during the 6 months prior to Screening<br>21. Reported using a Prohibited Medicine (excluding laxatives, suppositories, and enemas) during the Pretreatment Period or not willing or able to abide by the restrictions regarding use of Prohibited Medicines (Note: The use of fiber, bulk laxatives, or stool softeners [such as docusate] is acceptable provided the patient is on a stable dose during the 30 days before the Screening Visit and planned to continue on a stable dose throughout the trial).<br>22. Patients having following lab parameters deranged defined as follows:<br>• Renal function: Serum Creatinine $\geq 1.5 \times$ upper limit of normal (ULN)

	• Hepatic function: AST and ALT ≥3 × ULN;<br>• Hemoglobin: < 8g/dL<br>23. Has clinically significant laboratory findings or medical condition that could potentially affect study participation and/or personal well-being, as judged by the investigator.
**Randomization**	Subjects meeting the inclusion criteria and none of the exclusion criteria will be randomized to either of the following 2 arms.
**Dosing Schedule**	<u>Test Arm:</u> Elobixibat tablet<br>The usual adult dosage is 10 mg of Elobixibat administered orally once daily before breakfast. The dose may be adjusted according to the patient's response, but the maximum daily dose will be 15 mg.<br>Therefore, Elobixibat tablet (10 mg oral once daily administered as two tablets of 5 mg) in patients with chronic constipation once daily for one week and then dose titration (up or down) will be done according to the patient's response.<br><u>Comparator Arm:</u> Placebo matching test product<br>Placebo tablet matching test product (10 mg oral once daily administered as two tablets of 5 mg) in patients with chronic constipation once daily for one week and then dose titration of placebo (up or down) will be done according to the patient's response.
**Rescue Medicine**	Rescue Medicine, dispensed to patients, is a choice of 5-mg bisacodyl tablets or 10-mg bisacodyl suppositories.<br>During the Pretreatment and Treatment Periods, patients could use Rescue Medicine when at least 72

DocuSign Envelope ID: DCBE1147-372F-4B2F-B3C1-63958E711F1B

	hours had passed since their previous bowel movement or when their symptoms became intolerable.<br><br>A bowel movement will not be recorded in the analysis of efficacy if it occurs within 24 hours after the last administration of a rescue medication.<br><br>Data set prepared should include daily rescue medication used for each individual who used the rescue medication at any point during the study.
**Concomitant Medication**	Medications other than those mentioned in prohibited medications list will be permitted. The use of concomitant medication is based on the discretion of the Investigator. The Investigator may take the opinion of Medical Monitor to check for any probable drug interactions before taking the final decision on use of concomitant medication.<br><br>It should be clearly explained whether the medication was used prior to baseline visit, during the study, or both.
**Blinding**	The packaging of the test and placebo products should be similar in appearance to make differences in treatment less obvious to the subjects and to maintain adequate blinding of evaluators. Neither the subject nor the investigator should be able to identify the treatment. The containers should not be opened by the subject at the study center.
**Treatment duration**	2 Weeks
**Visits and study procedures**	Visit 1[#:] Screening visit Day -28 to Day -15<br><br># 2 weeks pretreatment period (Day -14 to Day -1) between screening and randomization

	Visit 2: Randomization visit, Day 0<br>Visit 3: Week 1+ (2) Days<br>Visit 4: Week 2+ (2) Days/ End of Study<br>• If a subject experiences any AE/SAE or worsening of the existing condition he/she can visit the site for an unscheduled visit. Subjects who withdraw from the study will be requested to attend an early termination visit, where Visit 4 procedures will be performed
**Criteria for Evaluation: Efficacy**	**Primary Endpoint**:<br>1. Change from baseline in weekly frequency of Spontaneous Bowel Movement (SBMs), Time Frame: From Baseline to 2 weeks.<br>• SBM is defined as the bowel movement occurring in the absence of laxative use or manual disimpaction in last 24 hours<br>**Secondary Endpoints:**<br>1. Proportion of patients with complete spontaneous bowel movement response (CSBM)<br>• CSBM is defined as SBM that is associated with a sense of complete evacuation.<br>• A CSBM responder is defined as a patient with ≥3 CSBMs per week and an increase of ≥1 CSBM per week from Baseline.<br>2. Proportion of patients with a SBM within 24 hour after the first dose of study drug<br>3. Median time to first SBM<br>• SBM is defined as a bowel movement that occurred in the absence of a laxative use or manual disimpaction in last 24 hours

	• Baseline value for the number of SBM is defined as the average of the numbers of SBM per week for over the 2-week pretreatment Period.<br>4. Stool consistency using Bristol Stool Form Scale (BSFS)<br>• Change from baseline in weekly Stool Consistency using BSFS score, Time Frame: From baseline to 2 weeks<br>• The stool consistency is measured using the seven-point ordinal BSFS score.<br>• The BSFS classifies human stool into seven types and points them accordingly.<br>o Type 1: Separate hard lumps, like nuts (hard to pass)<br>o Type 2: Sausage-shaped, but lumpy<br>o Type 3: Like a sausage but with cracks on its surface<br>o Type 4: Like a sausage or snake, smooth and soft<br>o Type 5: Soft blobs with clear cut edges (passed easily)<br>o Type 6: Fluffy pieces with ragged edges, a mushy stool<br>o Type 7: Watery, no solid pieces, entirely liquid<br>Types 1 and 2 indicate constipation, with 3 and 4 represents the ideal stool form (especially the latter), and 5, 6 and 7 tends towards diarrhoea.<br>• For a given assessment week, the weekly stool consistency is defined as the sum of non-missing stool consistency score for SBMs during that week divided

	by the number of non-missing stool consistency score for SBMs during that week.<br>5. Change From Baseline in Weekly Degree of Straining of SBMs<br>• The degree of straining is measured using the five-point ordinal scale (1=Not at all, 2=A little bit, 3=A moderate amount, 4=A great deal, and 5=An extreme amount).<br>• For a given assessment week, the weekly degree of straining is defined as the sum of non-missing straining score for SBMs during that week divided by the number of non-missing straining score for SBMs during that week.<br>6. Change from Baseline in Weekly Abdominal Bloating Score<br>• The abdominal pain score is measured using the five-point ordinal scale (1=None, 2=Mild, 3=Moderate, 4=Severe, and 5=Very severe).<br>• For a given assessment week, the weekly abdominal bloating score is defined as the sum of non-missing abdominal bloating score for SBMs during that week divided by the number of non-missing abdominal bloating score for SBMs during that week.<br>7. Change from Baseline in Weekly Abdominal Discomfort Score<br>• The abdominal discomfort score is measured using the five-point ordinal scale (1=None, 2=Mild, 3=Moderate, 4=Severe, and 5=Very severe).<br>• For a given assessment week, the weekly abdominal discomfort score is defined as the sum of non-missing

	abdominal discomfort score for SBMs during that week divided by the number of non-missing abdominal discomfort score for SBMs during that week.
**Criteria for Evaluation: Safety**	Safety evaluations (physical examination, laboratory investigation, vital signs, and ECG parameters, adverse events and serious adverse events) will be done during the study period. Adverse events will be documented if observed, mentioned during open questioning, or when spontaneously reported.<br><br>All adverse events (AEs) will be reported, whether or not they are considered to be related to the treatment. The report of AEs will include date of onset, description of the AE, severity, relation to study medication, action taken, outcome and date of resolution.
**Statistical Analysis Plan and Data handling/management**	A Statistical Analysis Plan (SAP) will be prepared as a separate document and finalized before database lock. Any deviation from the original statistical plan will be described and justified in the final report, as appropriate. The procedure for accounting for missing, unused and spurious data will be included in the Statistical Analysis Plan. All statistical analysis will be conducted using SAS®, Version 9.4 or higher.<br><br>Case Report Forms (CRFs) will be used to collect information required for statistical analysis. The CRF will be designed as per protocol, protocol amendment(s). Clinical Data Management team will set up the study database / application as specified in Data Management Plan. Data will be cleaned through query generation and resolution. Medical coding,

DocuSign Envelope ID: DCBE1147-372F-4B2F-B3C1-63958E711F1B

	handling of external data etc. will be done as per Data Management Plan. Medical coding will be done by using standard medical dictionaries like Med-DRA and WHODD.

LIST OF STUDY PERSONNEL:

Please refer to Section 18.2: Appendix 2

TABLE OF CONTENTS

TITLE 1

PROTOCOL VERSION HISTORY 3

Summary of Changes from Version 1.0 dated 11-MAR-2022 to Version 2.0 dated 17-OCT-2023 4

SIGNATURE PAGE 5

DECLARATION BY THE INVESTIGATOR 6

PROTOCOL SYNOPSIS 7

LIST OF STUDY PERSONNEL: 22

TABLE OF CONTENTS 23

1. INTRODUCTION 28

1.1 Background 28

1.2 Approved Product Information as per PMDA label 29

2. SCIENTIFIC RATIONALE OF THE STUDY 35

3. STUDY OBJECTIVES 37

3.1. Primary Objective 37

3.2. Secondary Objective 37

4. STUDY DESIGN & PLAN OF STUDY 38

4.1. Overview of the Study design 38

4.2. Flow chart of the study 41

4.3. Summary of Methods & Procedures 42

4.3.1. Efficacy Parameters: 42

4.3.2. Safety parameters 42

5. STUDY POPULATION 43

5.1. Inclusion Criteria 43

5.2. Exclusion Criteria 44

5.3. Withdrawal criteria 46

5.4. Rescue Medication 47

5.5. Planned Sample Size and Number of Study Centers 47

6. STUDY ASSESSMENTS 47

6.1. Efficacy assessments 47

6.2. Safety assessments 48

6.3. Efficacy Variables 48

6.3.1. Primary efficacy variables 48

6.3.2. Secondary Efficacy variables 48

7. STUDY CONDUCT 51

DocuSign Envelope ID: DCBE1147-372F-4B2F-B3C1-63958E711F1B

Table 1. Schedule of Events 51
7.1. Study Procedures 52
7.1.1. Visit 1 (Screening/ Day -28 to -1) 52
7.1.2. Visit 2 (Randomization visit/ Day 0) 53
7.1.3. Visit 3: Week 1/Day 7 (+2) 54
7.1.4. Visit 4: Week 2/Day 14 (+2)/ End of Treatment Visit: 54
7.2. Early Termination of the Study 56
7.3. Withdrawals and Dropouts 57
7.4. Protocol compliance and Protocol deviation 57
7.5. Audits and inspections 57
8. STUDY TREATMENT 58
8.1. Study Drug 58
8.2. Administration 58
8.3. Storage Requirements 58
8.4. Concomitant treatment 58
9. ADVERSE EVENTS 61
9.1. Adverse Events (clinical) 61
9.2. Adverse Events (laboratory and others) 61
9.3. Collection of Adverse Events 62
9.4. Definitions 62
9.5. Assessment of Adverse Events 62
9.5.1. Seriousness 62
9.5.2. Intensity (severity) 63
9.5.3. Causality assessment 65
9.6. Recording of Adverse Events 66
9.6.1. Procedures for Reporting of Adverse Events 66
9.7. Reporting of Serious Adverse Events 67
10. ETHICAL CONSIDERATIONS 72
10.1. Ethics Committee review and communications 72
10.2. Informed Consent Process 72
10.2.1. LAR (Legally Acceptable Representative) 73
10.2.2. Impartial Witness 73
10.2.3. Re-consenting Process 73
10.3. Subject Confidentiality 73
11. STUDY MONITORING 74
11.1. Monitoring Frequency and Agenda 74
11.2. Source Data Verification 74

11.3. Data Clarification / Rectification 74

11.4. Close-out Meeting / Site Closure 74

11.5. Trial Supplies Accounting & drug dispensing log........ 74

11.6. Records Preservation and Archiving 75

11.7. Subject Screening Log........ 75

11.8. Patient Identification Log 75

11.9. Informed Consent Forms 75

11.10. Source Documents........ 75

11.11. Study Documents........ 76

12. INVESTIGATIONAL PRODUCT MANAGEMENT 76

12.1. Study Drugs 76

12.2. Packaging, Labeling and Storage........ 76

12.3. Unused investigational products 77

12.4. Blinding and Unblinding Procedures........ 77

12.4.1. Randomization........ 77

12.4.2. Unblinding 77

12.4.2.1. Accidental Unblinding........ 78

12.4.2.2. Unblinding for Regulatory and Analysis requirement at the end of study…………...78

12.5. Supplies Accountability 78

13. STATISTICS and DATA ANALYSIS 78

13.1. Study Subjects........ 78

13.2. Sample Size Estimation 78

13.3. Statistical Analysis........ 79

13.3.1. Efficacy analysis........ 79

13.3.2. Safety Analysis 80

13.4. Data handling........ 80

14. QUALITY CONTROL AND QUALITY ASSURANCE 81

15. INVESTIGATOR RESPONSIBILITIES AND ESSENTIAL DOCUMENTS IN STUDY 81

16. FINAL STUDY REPORT 82

16.1. Publication Policy........ 82

17. REFERENCES 83

18. APPENDICES 84

18.1. Appendix 1: Declaration of Helsinki........ 85

18.2. Appendix 2: List of Study Personnel........ 93

18.3. Appendix 3. The Bristol Stool Form Scale (BSFS)........ 94

LIST OF ABBREVIATIONS AND DEFINITIONS OF TERMS

**Abbreviation**	**Full form**
AE	Adverse event/experience
AGA	American Gastroenterological Association
ANCOVA	Analysis of covariance
BM	Bowel Movement
BSFS	Bristol Stool Form Scale
CC	Chronic constipation
CBC	Complete blood count
CDM	Clinical Data Management
cGMP	Cyclic guanosine monophosphate
COA	Certificate of analysis
CRF	Case Report Form
CRO	Contract Research Organization
CSBM	Complete spontaneous bowel movement
DHHS	Department of Health and Human Services
ECG	Electrocardiogram
eCRF	Electronic case report form
EOT	End-of-Trial
FDA	US Food and Drug Administration
GC-C	Guanylate cyclase subtype C
GI	Gastrointestinal
IBS-C	Irritable bowel syndrome with constipation
ICF	Informed consent form
ICH	International Conference on Harmonization of Technical Requirements for Registration of Pharmaceuticals for Human Use
IRB	Institutional Review Board
ITT	Intent to treat
NA	Not applicable
PID	Patient identification
PK	Pharmacokinetic
SAE	Serious adverse event
SBM	Spontaneous bowel movement

SAS	Statistical analytics software.
TEAE	Treatment-emergent adverse event
UA	Urinalysis

1. INTRODUCTION

1.1 Background

The symptoms of chronic constipation are infrequent bowel movements, straining, sensation of incomplete evacuation, and hard stools.[1] These negatively affect quality of life (QOL) and impose a socioeconomic burden.[2] In the absence of rectal evacuation disorders, most patients with constipation have normal colonic transit but a few have slow colonic transit associated with reduced colonic propagated contractions.[3]

Chronic constipation (CC) is common among the elderly population. Many challenges are associated with the diagnosis and management of patients with CC[3]. Some of these challenges arise from the current incomplete understanding of what causes constipation and from the difficulties in diagnosing and classifying the heterogeneous group of patients with CC.

The prevalence of chronic constipation (CC) among adults is approximately 15%, making it the sixth most common gastrointestinal symptom. Chronic constipation often results in visits to ambulatory clinics and gastroenterology referrals.[4] Although the prevalence is greater in non-Caucasians than Caucasians, in women (median female to male ratio of 1.5:1), and in institutionalized rather than community-living elderly persons, symptoms can affect all ages, races, socioeconomic groups, and nationalities.

Constipation is labelled as ‘Chronic idiopathic constipation (CIC)’ when the cause cannot be identified. Global prevalence of CIC is 20%. Population based study from India in 505 people found the prevalence of constipation by the Rome II criteria to be 16.8% and self-reported constipation to be 24.8% in the last 1 year. Despite the availability of different treatment options for constipation, the quest for an unmet need for drugs in the treatment of patients with CC continues.[5]

Elobixibat is a novel, minimally absorbed inhibitor of ileal bile acid transporter (IBAT; also called apical sodium-dependent bile acid transporter), which is encoded by the gene SLC10A2 expressed locally in enterocytes. An IBAT inhibitor interrupts the enterohepatic circulation of bile acids (BA) and upregulates hepatic bile acid synthesis from cholesterol.[6] Upregulation of hepatic BA synthesis

promotes maintenance of BA pool size and may also lower serum LDL cholesterol concentrations.

Increased concentrations of bile acids in the colon enhance transit by dual actions: stimulating fluid and electrolyte secretion and inducing high-amplitude propagated contractions based on the effects of intraluminal chenodeoxycholate in the human colon.[7]

While constipation is treated with drugs such as irritant laxatives (eg, sennoside, sodium picosulfate hydrate), saline laxatives (eg, magnesium oxide), and drugs that alter intestinal epithelial function (eg, lubiprostone), as a single agent or in combination with other drugs, the long-term use of these drugs carries the risk of developing resistance or habituation, electrolyte abnormalities including hypermagnesemia, and nausea, respectively. Elobixibat suppresses the reabsorption of bile acids in the ileum by inhibiting the IBAT, and thereby increases the amount of bile acids in the colon. As a result, secretion of fluid and electrolytes in the intestinal tract and gastrointestinal motility are enhanced.

Elobixibat showed accelerated colonic transit, looser stool consistency, decreased constipation rating, and reduced straining compared with placebo in a Phase IIa study over 14 days. Elobixibat was associated with a significantly greater increase in the number of spontaneous bowel movements (SMBs) per week from baseline to placebo. Elobixibat also improves bloating severity.[8]

This study is planned to evaluate the efficacy, and safety of Elobixibat tablet (10 mg oral once daily administered as two tablets of 5 mg) in patients with chronic constipation once daily for one week and then dose titration (up or down) will be done according to the patient's response.

1.2 Approved Product Information as per PMDA label

Active ingredient: Elobixibat 5 mg in 1 tablet (5.13 mg as Elobixibat hydrate)

Additive: Crystalline cellulose, D-mannitol, hypromellose, sodium croscarmellose, light anhydrous silicic acid, magnesium stearate, macrogol 6000, titanium oxide, yellow iron sesquioxide, carnauba wax.

DocuSign Envelope ID: DCBE1147-372F-4B2F-B3C1-63958E711F1B

Elobixibat was jointly developed by EA Pharma and Mochida. EA Pharma and Mochida distribute elobixibat under the same brand name "GOOFICE 5mg Tablet", respectively, in Japan. [9]

Chemical name: [(2 R) -2- (2-{[3, 3-Dibutyl-7-(methylsulfanyl) -1,1-dioxo-5-phenyl-2,3,4,5-tetrahydro-1 H -1, 5-benzothiazepin-8-yl] oxy} acetamide) -2-phenylacetamido] acetic acid monohydrate.

Molecular formula: C 36 H 45 N 3 O 7 S 2 ・ H 2 O

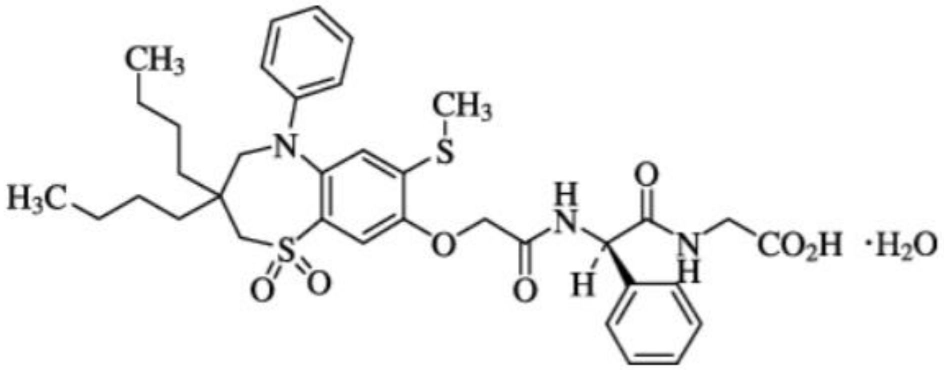

Properties: Elobixibat hydrate is a white powder. This product is freely soluble in N, N -dimethylformamide, sparingly soluble in acetonitrile or methanol, sparingly soluble in ethanol (99.5), and practically insoluble in water.

Clinical pharmacology:

Mechanism of Action:

Elobixibat inhibits the bile acid transporter (IBAT) expressed in the epithelial cells of the terminal ileum and suppresses the reabsorption of bile acids, and bile acids flowing into the colonic lumen. Increase the amount of. Bile acids secrete water and electrolytes into the large intestine lumen and further enhance gastrointestinal motility, so that the therapeutic effect of this drug on constipation is exhibited.

Pharmacodynamics:

In a rat constipation model induced by loperamide, Elobixibat showed a constipation-improving effect by a single oral administration.

DocuSign Envelope ID: DCBE1147-372F-4B2F-B3C1-63958E711F1B

Pharmacokinetics:

Absorption:

In 60 Japanese patients with chronic constipation, the effect on pharmacokinetics was evaluated by the crossover method depending on the presence or absence of food intake after a single oral administration of this drug. C max and AUC 0-∞ at the time of pre-meal administration were about 20 to 30% at the time of non-meal intake.

Food Effect:

When compared to fasting conditions, the plasma exposure of elobixibat decreased to 20-30% with breakfast. After repeated administration, plasma concentrations reached steady state after 7 days and no accumulation was found up to the dose of 15 mg. In clinical trials, Elobixibat was administered before breakfast.

Distribution:

The in vitro binding rate of elobixibat to human plasma protein was 99% or higher, and the human blood cell transfer rate was less than 5%.

Metabolism:

No metabolites were observed in the plasma of 6 healthy adult male foreigners who received a single oral dose of 14 C-elobixibat 5 mg (approximately 2.75 MBq). Unchanged and monohydroxyelobixibat were found in the pooled feces 24 to 48 hours after administration, but the radioactivity rates were 96.06% and 3.16%, respectively, and most were unchanged.

Excretion:

When a single oral dose of this drug was given to Japanese patients with chronic constipation under fasting conditions, the cumulative urinary drug excretion rate up to 144 hours after administration was about 0.01% of the dose, and most of the drug was excreted in the urine. Not recognized. When a single oral dose of 14 C-elobixibat 5 mg (about 2.75 MBq) was given to 6 healthy foreign male adults, 103.1% of the dose of radioactivity was excreted in the feces 144 hours after the administration, and urine was excreted. Radioactivity of 0.00 to 0.02% of the dose was excreted in it.

Precaution Regarding Specific Population:

Patients with severe liver damage: The effect of this drug may not be expected in patients with biliary obstruction or decreased bile acid secretion.

- Pregnant women: Administer to pregnant or potentially pregnant women only if the therapeutic benefit outweighs the risks. In animal experiments (rats), high-dose oral administration was found to affect maternal toxicity (1000 mg / kg / day) and the viability, growth and development of offspring (350 mg / kg / day or more).
- Lactating women: Consider continuing or discontinuing breastfeeding, taking into account the therapeutic benefits and benefits of breastfeeding. In animal experiments (rats) using C-elobixibat, the transfer of radioactivity into milk has been reported.
- Children, etc.: No clinical trials have been conducted for children.
- Elderly: Be careful, such as losing weight. In general, the physiological function is often deteriorated.

<u>Drug Interaction Studies:</u>

The IC 50 value of elobixibat for the transport of digoxin, which is a substrate for P-glycoprotein, in Caco-2 cells was 2.65 μmol / L, indicating an inhibitory effect on P-glycoprotein. Twenty-five foreign healthy adult males and females were orally administered 10 mg of this drug once daily for 5 days, with dabigatran etexilate 150 mg / dose / day on the first day, midazolam 2 mg / dose / day on the first day, and It was used in combination on the 5th day and compared with each single administration. As a result, the AUC 0-t and C max of dabigatlan, which is a substrate for P-glycoprotein, were 1.17 times (90% confidence interval: 1.00-1.36) and 1.13 times (90% confidence interval), respectively, compared with the single administration. : 0.96-1.33), and the upper limit of the 90% confidence interval exceeded the standard value of 1.25. AUC 0-t and C max on day 5 of Midazolam were 0.78 times (90% confidence interval: 0.73-0.83) and 0.94 times (90% confidence interval: 0.87-1.01), respectively, compared to the single administration.

The lower limit of the 90% confidence interval for AUC 0-t fell below the reference value of 0.80 4). This drug has an inhibitory effect on P-glycoprotein.

Precautions for combined use (Be careful about combined use):

**Drug name, etc.**	**Clinical symptoms / measures**	**Mechanism / risk factors**
Bile acid preparation<br>Ursodeoxycholic acid, chenodeoxycholic acid	The effects of these agents may be diminished.	The bile acid transporter (IBAT) inhibitory action of this drug may inhibit the reabsorption of bile acid preparations.
Aluminum containing antacid Sucralfate hydrate, aldioxa, etc	The action of this drug may be diminished.	Since these drugs adsorb bile acids in the gastrointestinal tract, the action of this drug may be diminished.
Cholestyramine, cholestyramine	The action of this drug may be diminished.	Since these drugs adsorb bile acids, the action of this drug may be diminished.
Digoxin, dabigatran etexilate methanesulphonate	Blood levels of these drugs may increase and their effects may be enhanced.	Due to the inhibitory effect of this drug on P-glycoprotein.
Midazolam	The blood concentration of midazolam may decrease and its action may be diminished.	The mechanism is unknown

Side effects:

The following side effects may occur, so observe carefully and take appropriate measures such as discontinuing administration if any abnormalities are observed.

	5% or more	Less than 1- 5%	Less than 1%	Frequency unknown
Liver		Liver dysfunction (ALT increased, AST increased, γGTP increased, AlP increased, LAP increased)		
Psycho-nervous system		Floating dizziness		Headache
Cardiovascular				Hot flashes
Digestive organ	Abdominal pain (23.2%), diarrhea (14.4%)	Lower abdominal pain, bloating, nausea, upper abdominal pain, abdominal discomfort, loose stools.	Mouth ulcer, dry mouth	Flatulence, urgency, vomiting, abnormal gastrointestinal sounds, constipation
Hypersensitivity			Urticaria	rash
blood		Anemia	Increased vitamin E	Increased eosinophil count
others		CK increase		Dysmenorrhea

Contraindications:

- Patients with a history of hypersensitivity to the ingredients of this drug
- Patients with confirmed or suspected intestinal obstruction due to tumor, hernia, etc. [May worsen intestinal obstruction].

DocuSign Envelope ID: DCBE1147-372F-4B2F-B3C1-63958E711F1B

2. SCIENTIFIC RATIONALE OF THE STUDY

The clinical development program for Elobixibat concluded results from two phase 3 clinical trials (a 2-week double-blind placebo-controlled phase 3 trial and an open-label single-arm 52-week long-term phase 3 trial) for the bile acid transporter inhibitor "GOOFICE 5mg Tablet" (nonproprietary name: elobixibat hydrate; development code: AJG533, hereinafter "elobixibat") was published in The Lancet Gastroenterology & Hepatology) a journal of The Lancet.[10]

The 2-week double-blind clinical trial was a placebo-controlled, randomized, double-blind trial with 133 Japanese patients with chronic constipation. Patients were orally administered 10 mg of elobixibat or placebo once daily for 2 weeks. Eligible patients were Japanese men and non-pregnant women aged 20–80 years, with chronic constipation of at least 6 months' duration, diagnosed on the basis of standard symptom-based criteria of fewer than three spontaneous bowel movements per week (defined as bowel movements occurring spontaneously and independently of administration of rescue medication for at least 24 h), with at least one of the following symptoms during 25% or more of bowel movements: straining, lumpy or hard stools, and sensation of incomplete evacuation. These criteria satisfied Rome III criteria for functional constipation.[7]

The elobixibat group demonstrated statistically significant improvements in the primary endpoint of change in SBM frequency, as well as in secondary endpoints including change in complete spontaneous bowel movement (CSBM) frequency (the secondary endpoint), length of time between dosing and the first spontaneous bowel movement, compared to the placebo group. Results of a phase 2b trial.[8] in Japanese patients with chronic constipation revealed that 10 mg of elobixibat once per day was safe and effective; hence, 10 mg was selected for a short-term, phase 3 trial of safety and efficacy to confirm the findings from the phase 2b trial.

A 2-week, phase 2b trial[8] in Japanese patients with chronic constipation showed that 10 mg of Elobixibat once per day was safe and effective; hence, 10 mg was selected for a short-term, phase 3 trial of safety and efficacy to confirm the findings from the phase 2b trial.

The present study is planned to evaluate the efficacy, and safety of Elobixibat tablet (10 mg oral once daily administered as two tablets of 5 mg) in Indian patients with

DocuSign Envelope ID: DCBE1147-372F-4B2F-B3C1-63958E711F1B

chronic constipation once daily for one week and then dose titration (up or down) will be done according to the patient's response.

DocuSign Envelope ID: DCBE1147-372F-4B2F-B3C1-63958E711F1B

3. STUDY OBJECTIVES

3.1. Primary Objective

To evaluate the efficacy of Elobixibat tablet in patients with chronic constipation.

3.2. Secondary Objective

To evaluate the safety and tolerability of Elobixibat tablet in patients with chronic constipation.

4. STUDY DESIGN & PLAN OF STUDY

4.1. Overview of the Study design

Study Type: Interventional

Study Design: Prospective, multicenter, randomized, double blind, parallel group, placebo controlled, phase III superiority study

Randomization: Subjects meeting the inclusion criteria and none of the exclusion criteria will be randomized to either of the 2 arms i.e. test arm or placebo arm.

The packaging of the test and placebo products will be similar in appearance to make differences in treatment less obvious to the subjects and to maintain adequate blinding of evaluators. Neither the subject nor the investigator will be able to identify the treatment. The containers will not be opened by the subject at the study center.

Method of Generating Random Sequence: Computer generated randomization.

Method of Concealment: Sequentially numbered, sealed, opaque envelopes.

Blinding/Masking: Double Blind

Dosing Schedule:

- Test Arm: Elobixibat tablet

The usual adult dosage is 10 mg of Elobixibat self-administered orally once daily before breakfast. The dose may be adjusted according to the patient's response, but the maximum daily dose is 15 mg. Therefore, Elobixibat tablet (10 mg oral once daily self-administered as two tablets of 5 mg) in patients with chronic constipation once daily for one week and then dose titration (up or down) will be done according to the patient's response.

- Comparator Arm: Placebo matching test product

Placebo tablet matching test product (10 mg oral once daily self-administered as two tablets of 5 mg) in patients with chronic constipation once daily for one week and then dose titration of placebo (up or down) will be done according to the patient's response.

Trial visits:

Visit 1# (Screening visit Day -28 to Day -15)

2 weeks pretreatment period (Day -14 to Day -1) between screening and randomization

Visit 2 (Randomization visit, Day 0)

Visit 3: Week 1/ Day 7 (+2)

Visit 4: Week 2/Day 14 (+2)

Treatment Group	Drug Administered	No of Subjects
Intervention/Test Product	Elobixibat 5 mg tablet.*	75
Comparator	Placebo (Matching placebo oral once daily as two tablets)	75

* Elobixibat tablet (10 mg oral once daily self-administered as two tablets of 5 mg) in patients with chronic constipation once daily for one week and then dose titration (up or down) will be done according to the patient's response.

The trial consists of three distinct periods defined as follows:

1. **The screening period** starts with signature of the ICF and lasts for up to 28 calendar days. During this period, patient eligibility for entry into the pretreatment period will be determined. This study includes a 2-week pre-treatment period (day -14 to -1) between screening and randomization to assess the study eligibility and baseline SBM frequency. Assessment of prohibited medications is conducted at the time of screening and accordingly if the patient meets the entry criteria assessed at the Screening Visit and does not require a washout period then patient can directly enter the pretreatment period of 14 days after screening. If the patient requires a washout period, then pretreatment period is started after the completion of wash out period. Any over-the-counter or prescription laxatives, suppositories, or enemas used to treat Chronic Constipation must not be taken during the calendar day before the start of Pretreatment period; whereas other prohibited medicines must not be taken during the 14 calendar days before the start of Pretreatment period. ***The pretreatment period*** is defined as the 14 calendar days immediately before randomization.

Note: Patients reporting more than 3 SBM during pretreatment period will be excluded from the study.

2. **The treatment period** begins with randomization and lasts for 2 weeks. Patients who meet all entry criteria will be randomized to treatment with 5*mcg Elobixibat, or placebo (1:1).

* Elobixibat tablet (10 mg oral once daily self-administered as two tablets of 5 mg) in patients with chronic constipation once daily for one week and then dose titration (up or down) will be done according to the patient's response.

Patient will be advised to take study drug once daily in the morning before breakfast from Day 1.

Patients will continue to provide their daily assessments, their weekly assessments, and their use of rescue medicine and any other laxatives, suppositories, or enemas up to 2 week or EOT.

A list of these assessments and the visits when they are performed is provided in the Schedule of Evaluations.

4.2. Flow chart of the study

Study overview:

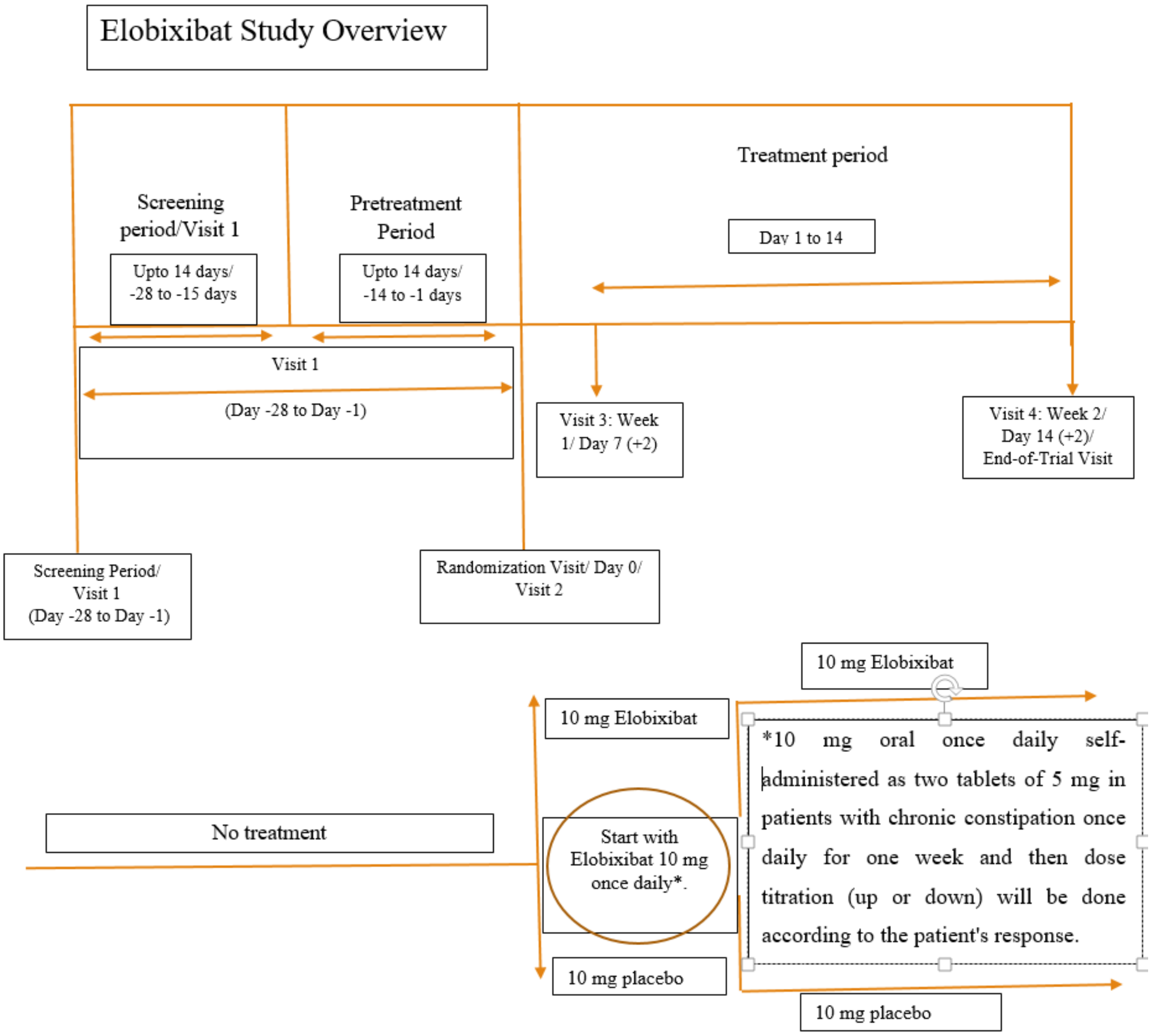

4.3. Summary of Methods & Procedures

4.3.1. Efficacy Parameters:

Primary Efficacy Endpoints:

1. Change from baseline in weekly frequency of Spontaneous Bowel Movement (SBMs), Time Frame: From Baseline to 2 weeks.

Secondary Efficacy Endpoints:

1. Proportion of patients with complete spontaneous bowel movement (CSBM)
2. Proportion of patients with a SBM within 24 h after the first dose of study drug
3. Median time to first SBM
4. Change from baseline in Stool consistency using Bristol Stool Form Scale (BSFS)
5. Change from Baseline in Weekly Degree of Straining of SBMs
6. Change from Baseline in Weekly Abdominal Bloating Score
7. Change from Baseline in Weekly Abdominal Discomfort Score

#An SBM is a BM that occurs in the absence of laxative, suppository, or enema use 24 hour of the BM or the calendar day before the BM

#A CSBM is an SBM that is associated with a sense of complete evacuation.

4.3.2. Safety parameters

All adverse events (AEs) should be reported whether or not they are considered to be related to the treatment. The report of AEs should include date of onset, description of the AE, severity, relation to study medication, action taken, outcome and date of resolution.

5. STUDY POPULATION

5.1. Inclusion Criteria

Subjects will be enrolled in the study if they meet all the following criteria.

1. Male or female patients of 18-65 years age
2. Ambulatory patients with chronic constipation (fulfilling Rome IV criteria) reporting <3 Spontaneous Bowel Movements (SBM) per week and reports one or more of the following symptoms for the last 3 months with symptom onset at least 6 months before the Screening Visit or before starting chronic therapy with any laxative:
   - Straining during more than 25% of defecations
   - Lumpy or hard stools more than 25% of defecations
   - Sensation of incomplete evacuation more than 25% of defecations
3. Patients with less than 3 SBM per week during pretreatment period.
4. Willing to comply with protocol requirements and sign a statement of informed consent
5. Willing to discontinue any laxatives used before the Pretreatment period in favor of the protocol-defined Rescue Medicine (bisacodyl tablets or suppositories)
6. Agreed to refrain from making any new, major life-style changes that may have affected CIC symptoms (e.g., starting a new diet or changing his or her exercise pattern) from the time of signature of the ICF to the last trial visit.
7. Female subjects of:

   Child-bearing potential should have negative serum pregnancy test at screening and agree to use adequate birth control during the entire study period (acceptable methods include intrauterine device, barrier or abstinence).

   OR

   Is surgically sterile (bilateral tubal ligation, bilateral oophorectomy, or hysterectomy has been performed on the study subject).

   OR

Postmenopausal for at least 1 year

5.2. Exclusion Criteria

1. Pregnant, breast feeding, or planning a pregnancy
2. Subject of any age with evidence of clinically significant weight loss, anemia, or rectal bleeding and without documentation of the results of either a flexible sigmoidoscopy or colonoscopy performed within 2 years period prior to screening.

   Note: Colonoscopy can be avoided for patients age less than 45 years and those who do not have alarming symptoms of weight loss, rectal bleeding or anemia in past 6 months based on Investigator discretion/ Medical Monitor opinion.
3. Reports loose (mushy) or watery stools in the absence of any laxative intake in the form of a tablet, a suppository or an enema, or prohibited medicine for >25% of bowel movements.
4. Reported a Bristol Stool Form Scale score of 6 (loose, mushy stools) for > 1 SBM or a Bristol Stool Form Scale score of 7 (watery stools) with any SBM over the 2 weeks pretreatment period before the Randomization Visit [*Refer to annexure 3 for BSFS score*].
5. Received a diagnosis of inflammatory bowel disease (IBD)
6. Meet the Rome IV criteria for Irritable Bowel Syndrome (IBS) or the Rome IV criteria for Opioid-Induced Constipation (OIC) as described below:

Rome Criteria for IBS and OIC

**IRRITABLE BOWEL SYNDROME**<br>*Diagnostic criteria**	**OPIOID-INDUCED CONSTIPATION**<br>*Diagnostic criteria*
Recurrent abdominal pain on average at least 1 day/week in the last 3 months, associated with two or more of the following criteria:	1. New, or worsening, symptoms of constipation when initiating, changing, or increasing opioid therapy, that must include two or more of the following:
Related to defecation	• Straining during more than ¼ (25%) of defecations
Associated with a change in frequency of stool	• Lumpy or hard stools (Bristol Stool Form Scale 1-2) more than ¼ (25%) of defecations
Associated with a change in form (appearance) of stool	2. Sensation of incomplete evacuation more than ¼ (25%) of defecations
	3. Sensation of anorectal obstruction/blockage more than ¼ (25%) of defecations
* Criteria fulfilled for the last 3 months with symptom onset at least 6 months prior to diagnosis	4. Manual maneuvers to facilitate more than ¼ (25%) of defecations (e.g., digital evacuation, support of the pelvic floor)

DocuSign Envelope ID: DCBE1147-372F-4B2F-B3C1-63958E711F1B

	5. Fewer than three SBM per week<br>6. Loose stools are rarely present without the use of laxatives.

IBS: Irritable Bowel Syndrome, OIC: Opioid Induced Constipation, SBM: Spontaneous Bowel Movement

7. Has a history of structural abnormality of the Gastrointestinal (GI) tract or a disease or condition that can affect GI motility.
8. Has a history of diverticulitis, chronic pancreatitis, active peptic ulcer disease (PUD) not adequately treated, ischaemic colitis, inflammatory bowel disease, laxative abuse, faecal impaction that required hospitalization or emergency treatment, pseudo-obstruction, mega colon, mega rectum, bowel obstruction, descending perineum syndrome, ovarian cysts, endometriosis, solitary rectal ulcer syndrome, systemic sclerosis, pre-malignant colonic disease (e.g., familial adenomatous polyposis or hereditary non-polyposis colorectal cancer) or other forms of familial colorectal cancer.
9. Diagnosis or family history of familial adenomatous polyposis, hereditary nonpolyposis colorectal cancer, or any other form of familial colorectal cancer
10. Current active peptic ulcer disease (i.e., disease that was not adequately treated or stable with therapy)
11. Has unexplained and clinically significant GI alarm signals (e.g., lower GI bleeding or heme-positive stool in the absence of known internal or external haemorrhoids, iron-deficiency anaemia, unexplained weight loss) or systemic signs of infection or colitis
12. Has a potential central nervous system (CNS) cause of constipation (e.g., Parkinson's disease, spinal cord injury, multiple sclerosis)
13. Has intestinal/rectal prolapse or other known pelvic floor dysfunction
14. Commonly uses digital maneuvers (perianal pressure or digital disimpaction) or vaginal splinting to facilitate the passage of a bowel movement
15. Has a history of diabetic neuropathy
16. Bariatric surgery for treatment of obesity, or surgery to remove a segment of the GI tract at any time before the Screening Visit, surgery of the abdomen, pelvis,

or retroperitoneal structures during the 6 months before the Screening Visit, an appendectomy or cholecystectomy during the 60 days before the Screening Visit, other major surgery during the 30 days before the Screening Visit.

17. Has a history of cancer with last date of proven disease activity/presence of malignancy within 5 years, except for adequately treated basal cell carcinoma of the skin, cervical dysplasia, or carcinoma in situ of the skin or the cervix
18. Known human immunodeficiency virus (HIV) or Hepatitis B/C (HBV/HCV) infection
19. Has a history of hospitalization for any psychiatric disorder, or any suicide attempt in the 2 years prior to Screening
20. History of alcohol or drug abuse during the 6 months prior to Screening
21. Reported using a Prohibited Medicine (excluding laxatives, suppositories, and enemas) during the Pretreatment Period or was not willing or able to abide by the restrictions regarding use of Prohibited Medicines (Note: The use of fiber, bulk laxatives, or stool softeners [such as docusate] was acceptable provided the patient had been on a stable dose during the 30 days before the Screening Visit and planned to continue on a stable dose throughout the trial).
22. Patients having following lab parameters deranged defined as follows:
    - **Renal function:** Serum Creatinine ≥1.5 × upper limit of normal (ULN)
    - **Hepatic function:** AST and ALT ≥3 × ULN;
    - **Hemoglobin:** < 8g/dL
23. Has a clinically significant laboratory finding or medical condition that could potentially affect study participation and/or personal well-being, as judged by the investigator.

5.3. Withdrawal criteria

- A subject may be withdrawn from therapy
- On account of development of an intolerable adverse event.
- A female subject will be withdrawn if she gets pregnant during the course of the study.

- A subject may also be withdrawn based on the discretion of the investigator for any reason, if it is felt that his/her further continuation in the trial will adversely affect him/her, or, in the interests of the study.
- Subject will be considered to have withdrawn from the study if the subject is lost to follow-up, died, is no longer being followed at the investigator's discretion
- A subject may also be withdrawn if the subject volunteers to do so. The reason for discontinuation should be documented and all serious adverse events should be reported to the Medical Monitor from Dr. Reddy's Laboratories.

All the efficacy and safety parameters are to be assessed and all applicable activities of visit 4 will be conducted at the time of discontinuation of the study.

The primary reason for withdrawing from the study will be clearly documented in the subject's medical record and recorded on the CRF.

5.4. Rescue Medication

Rescue Medicine, dispensed to patients, is a choice of 5-mg bisacodyl Capsules or 10-mg bisacodyl suppositories.

During the Pretreatment and Treatment Periods, patients could use Rescue Medicine when at least 72 hours had passed since their previous bowel movement or when their symptoms became intolerable.

A bowel movement will not be recorded in the analysis of efficacy if it occurs within 24 hour after the last administration of a rescue medication. Data set prepared should include daily rescue medication used for each individual who used the rescue medication at any point during the study.

5.5. Planned Sample Size and Number of Study Centers

A total of 150 subjects will be enrolled in this study from approximately 10-15 study centres across India.

6. STUDY ASSESSMENTS

6.1. Efficacy assessments

1. Change from baseline in weekly frequency of Spontaneous Bowel Movement

DocuSign Envelope ID: DCBE1147-372F-4B2F-B3C1-63958E711F1B

(SBMs)

2. Proportion of patients with complete spontaneous bowel movement (CSBM)
3. Proportion of patients with a SBM within 24 hour after the first dose of study drug
4. Median time to first SBM
5. Change from baseline in Stool consistency using Bristol Stool Form Scale (BSFS)
6. Change from Baseline in Weekly Degree of Straining of SBMs
7. Change from Baseline in Weekly Abdominal Bloating Score
8. Change from Baseline in Weekly Abdominal Discomfort Score

6.2. Safety assessments

The safety parameters include AEs, clinical laboratory parameters, vital signs, and ECG parameters. For each safety parameter, the last assessment made before randomization will be used as the baseline parameter.

6.3. Efficacy Variables

6.3.1. Primary efficacy variables

1. Change from baseline in weekly frequency of Spontaneous Bowel Movement (SBMs)
   - SBM is defined as bowel movement occurring in the absence of laxative use or manual disimpaction in last 24 hours
   - Change from baseline in the frequency of SBM per week.

6.3.2. Secondary Efficacy variables

1. Proportion of patients with complete spontaneous bowel movement (CSBM)
   - CSBM is defined as SBM that is associated with a sense of complete evacuation.
   - A CSBM responder is defined as a patient with ≥3 CSBMs per week and an increase of ≥1 CSBM per week from Baseline.

2. Proportion of patients with a SBM within 24 h after the first dose of study drug
3. Median time to first SBM

    - SBM is defined as a bowel movement that occurred in the absence of a laxative use or manual disimpaction.
    - Baseline value for the number of SBM is defined as the average of the numbers of SBM per week for over the 2-week pretreatment Period.

4. Change from baseline in Stool consistency using Bristol Stool Form Scale (BSFS)

- Change from baseline in weekly Stool Consistency using BSFS score, Time Frame: From baseline to 2 weeks
- The stool consistency is measured using the seven-point ordinal BSFS score.
- The BSFS classifies human stool into seven types and points them accordingly:
    - Type 1: Separate hard lumps, like nuts (hard to pass)
    - Type 2: Sausage-shaped, but lumpy
    - Type 3: Like a sausage but with cracks on its surface
    - Type 4: Like a sausage or snake, smooth and soft
    - Type 5: Soft blobs with clear cut edges (passed easily)
    - Type 6: Fluffy pieces with ragged edges, a mushy stool
    - Type 7: Watery, no solid pieces, entirely liquid
- Types 1 and 2 indicate constipation, with 3 and 4 represents the ideal stool form (especially the latter), and 5, 6 and 7 tends towards diarrhoea.
- For a given assessment week, the weekly stool consistency is defined as the sum of non-missing stool consistency score for SBMs during that week divided by the number of non-missing stool consistency score for SBMs during that week.

5. Change from Baseline in Weekly Degree of Straining of SBMs

- The degree of straining is measured using the five-point ordinal scale (1=Not at all, 2=A little bit, 3=A moderate amount, 4=A great deal, and 5=An extreme amount).
- For a given assessment week, the weekly degree of straining is defined as the sum of non-missing straining score for SBMs during that week divided by the number of non-missing straining score for SBMs during that week.

6. Change from Baseline in Weekly Abdominal Bloating Score

- The abdominal pain score is measured using the five-point ordinal scale (1=None, 2=Mild, 3=Moderate, 4=Severe, and 5=Very severe).
- For a given assessment week, the weekly abdominal bloating score is defined as the sum of non-missing abdominal bloating score for SBMs during that week divided by the number of non-missing abdominal bloating score for SBMs during that week.

7. Change from Baseline in Weekly Abdominal Discomfort Score

- The abdominal discomfort score is measured using the five-point ordinal scale (1=None, 2=Mild, 3=Moderate, 4=Severe, and 5=Very severe).
- For a given assessment week, the weekly abdominal discomfort score is defined as the sum of non-missing abdominal discomfort score for SBMs during that week divided by the number of non-missing abdominal discomfort score for SBMs during that week.

7. STUDY CONDUCT

Table 1. Schedule of Events

Visit	Visit 1<br>Screening^<br>Day -28 to Day -1	Visit 2, Day 0<br>Baseline/<br>Randomization	Visit 3/<br>Week 1<br>Day 7 (+2)	Visit 4 (End of the study)/ Week 2<br>Day 14 (+2)
**Consent**	X			
**Weight and height**	X			
**Vitals**	X	X	X	X
**Medical history**	X			
**Concomitant medication**	X	X	X	X
**Physical examination$**	X			X
**12-lead ECG**	X			
**Chest X-ray#**	X			
**Colonoscopy***	X			
**Pregnancy test (female patients of child bearing potential)**	X (Serum)	X (Urine)	X (Urine)	X (Urine)
**Haematology/Biochemistry/Urine analysis$**	X			X
**Inclusion/exclusion criteria**	X			
**Inclusion and Exclusion criteria Verification**		X		
**Randomization**		X		
**Study Drug Dispensing**		X	X	
**Study Drug return and accountability check**			X	X
**Subject Diary Dispensing**	X[a]	X	X	
**Subject Diary collection and Review**		X	X	X
**SBM Assessment**	X	X	X	X
**Assessment of CSBM, Stool consistency, Degree of Straining, Abdominal bloating, Abdominal discomfort**	X	X	X	X
**Adverse events recording**	X	X	X	X

^This study includes a 2-week pre-treatment period (day -14 to -1) between screening and randomization to assess the study eligibility and baseline SBM frequency. *Patients reporting more than 3 SBM during pretreatment period will be excluded from the study.*

$ Physical examination includes Cardiovascular, Nervous, Gastrointestinal, Respiratory system and any other specific examination, as per the investigator's discretion.

#X-ray done in past 3 months of screening will be valid;

*Colonoscopy reports of within two years period from screening time are valid; Colonoscopy can be avoided for patients age less than 45 years and those who do not have alarming symptoms of weight loss, rectal bleeding or anemia in past 6 months based on Investigator discretion/ Medical Monitor opinion (Rectal examination to be performed during the screening period on all patients who do not require a colonoscopy). SBM: Spontaneous Bowel Movement; CSBM: Complete Spontaneous Bowel Movement;

If the patient meets the entry criteria assessed at Screening and requires a washout period according to prohibited medicine section as per protocol, below mentioned points should be followed:

$Lab assessments including Haematology/Biochemistry/Urine analysis will be conducted after completion of 14 day wash out period. This will be considered as part of the screening visit.

[a] Diary entry should start after the completion of wash out period:

If the patient meets the entry criteria assessed at the Screening Visit and does not require a washout of prohibited medicines, then patient can be randomized after completion of 2 weeks pretreatment period and diary and rescue medications can be provided on the same day

7.1. Study Procedures

7.1.1. Visit 1 (Screening/ Day -28 to -1)

At the Screening Visit, a review of inclusion and exclusion criteria will be conducted to determine the patient's eligibility for progression to the pretreatment period. Trial procedures will be reviewed with the patient, the caregiver, and/or the legally authorized representative (if different from the caregiver); and documentation of informed consent will be obtained. After the patient signs the ICF, the Trial Coordinator will register the patient for the screening period, and the patient will then be assigned a unique PID number in sequential order. The following procedures will be performed thereafter:

- Recording of medical history
- Performance of physical examination (Cardiovascular, Nervous, Gastrointestinal, Respiratory system and any other specific examination, as per the investigator's discretion).
- Measurement of body weight and height
- Recording of vital signs (temperature, respiratory rate, systolic and diastolic blood pressure, and pulse rate)
- Chest X-ray#

  # X-ray done in past 3 months of screening will be valid
- 12-lead ECG
- Rectal examination should be performed during the screening period on all patients who do not require a colonoscopy. Colonoscopy can be avoided for patients age less than 45 years and those who do not have alarming symptoms of weight loss, rectal bleeding or anemia in past 6 months based on Investigator discretion/ Medical Monitor opinion. In subsequent visits, the rectal examination may be performed at the discretion of the Investigator
- Documentation of prior and concomitant medicines
- Collection of blood and urine samples for clinical laboratory determinations* (Refer table 2 for details of laboratory investigations)

  **if the patients are required to undergo 14 days wash out period then these investigations to be conducted after completion of wash out period.*
- Adverse event (AE) assessment
- Providing instructions for washout period, if required
- Review of inclusion and exclusion criteria
- Diary training and dispensing of rescue medication#

#if the patients are required to undergo 14 days wash out period then these activities to be conducted after completion of wash out period

- Providing all applicable instructions to the patients regarding pretreatment period
- Two weeks pretreatment period (day -14 to -1) is in between screening and randomization
- The purpose of this pretreatment period is to collect information regarding bowel habits of patients through patient diaries and to determine whether the patient is eligible to continue into the treatment period of the trial.

7.1.2. Visit 2 (Randomization visit/ Day 0)

The following procedures will be performed before randomization and administration of the first dose of study drug at the trial center:

- Recording of vital signs (temperature, respiratory rate, systolic and diastolic blood pressure, and pulse rate)
- Diary Collection and review of Patient Diary to assess:
  - Number of Spontaneous Bowel Movements
  - Sensation of complete evacuation
  - Stool consistency
  - Severity of straining
  - Abdominal Bloating Score
  - Abdominal Discomfort Score
  - Complete Spontaneous Bowel Movements
  - Use of per-protocol rescue medicine

- Urine pregnancy test for females of childbearing potential
- Review of adverse events (AEs)
- Verification of inclusion and exclusion criteria
- Documentation of concomitant medicines used if any
- Allocation of the treatment as per randomization
- Dispensing of study drug
- Dispensing of rescue medication (if required)
- Issue of subject Diary with instructions

7.1.3. Visit 3: Week 1/Day 7 (+2)

The following procedures will be performed:

- Vital signs

Diary Collection and review of Patient Diary for:

  - Number of Spontaneous Bowel Movements
  - Sensation of complete evacuation
  - Stool consistency
  - Severity of straining
  - Abdominal Bloating Score
  - Abdominal Discomfort Score
  - Complete Spontaneous Bowel Movements
  - Use of per-protocol rescue medicine

- Urine pregnancy test for females of childbearing potential
- Review of adverse events (AEs)
- Documentation of concomitant medicines used if any
- Study drug return and accountability check
- Dispensing of study drug
- Dispensing of rescue medication (if required)
- Issue of subject Diary with instructions

7.1.4. Visit 4: Week 2/Day 14 (+2)/ End of Treatment Visit:

Any clinical findings obtained during the final examination or at premature discontinuation for any reason, including clinically significant laboratory abnormalities, will be followed until the condition returns to screening status, has resolved or stabilized, or can be explained as being unrelated to study drug.

Patients who complete the Treatment Period will undergo the following procedures:

- Physical examination
- Vital signs
- 12-Lead ECG

Diary Collection and review of Patient Diary for:

  - Number of Spontaneous Bowel Movements
  - Sensation of complete evacuation
  - Stool consistency

- Severity of straining
- Abdominal Bloating Score
- Abdominal Discomfort Score
- Complete Spontaneous Bowel Movements
- Use of per-protocol rescue medicine

- Urine pregnancy test for females of childbearing potential
- Review of adverse events (AEs)
- Documentation of concomitant medicines used if any
- Study drug return and accountability check
- Return of rescue medication
- Collection of blood and urine samples for clinical laboratory determinations. Refer table for details of laboratory investigations.

Clinical Laboratory Determination:

At the Screening Visit (Visit 1) and End-of-Trial visit, the Investigator will assess clinical significance of any values outside the reference ranges.

The following clinical laboratory tests will be performed:

Table 2: Clinical Laboratory Tests

Hematology	Biochemistry	Urinalysis
• Hemoglobin (Hb %)<br>• Total and Differential WBC count<br>• Platelet count<br>• RBC count<br>• Absolute Neutrophil Count	• RBS<br>• **Renal Function Test:**<br>Blood urea nitrogen, Creatinine<br>• **Liver Function Test**<br>Total Protein, S. albumin,<br>Total and direct bilirubin, ALT, AST, Alkaline phosphatase<br>• **Serum electrolytes:**<br>Sodium, Potassium and chloride	Colour<br>Transparency<br>pH<br>Specific gravity<br>Protein<br>Glucose<br>Ketone bodies<br>Bilirubin<br>Blood<br>Nitrite<br>Urobilinogen<br>**Microscopic examination**<br>WBC, RBC, Casts, Crystals
• Serum Pregnancy test#<br>• Urine Pregnancy Test (UPT)*		
# Serum pregnancy test will be performed at screening visit/Visit 1		
* Urine pregnancy will be performed at every subsequent visit after screening visit		

ALT=alanine transaminase, AST=aspartate aminotransferase, RBC=red blood cell, RBS=random blood sugar, WBC=white blood cells

7.2. Early Termination of the Study

Sponsor reserves right to discontinue the study at any time. Principal Investigator reserves the right to discontinue the study for safety reasons at any time. Independent / Institutional Ethics Committee (IEC) may ask to terminate the study, if there are major violations of ethical considerations or due to any serious adverse event(s). Reason(s) for termination of study will be provided to the subjects.

If a subject experiences any AE/SAE or worsening of the existing condition he/she can visit the site for an unscheduled visit. Subjects who withdraw from the study will be requested to attend an early termination visit, where Visit 4 (end of trial visit) procedures will be performed.

7.3. Withdrawals and Dropouts

Please refer the section 5.3 (Withdrawal Criteria) of the protocol for details of withdrawal criteria.

7.4. Protocol compliance and Protocol deviation

The study shall be conducted in compliance with the protocol agreed to by the sponsor and as per approval by the regulatory authority(ies) and which was given approval/favourable opinion by the IEC.

The investigator should not implement any deviation from, or changes of the protocol without agreement by the sponsor and prior review and documented approval/favourable opinion from the IEC of an amendment, except where necessary to eliminate an immediate hazard(s) to study subjects.

While deviations from the protocol should be avoided, if deviations do occur, the investigator/site staff must inform the sponsor monitor and the implications of deviations will be reviewed and discussed. Deviations will be documented and explained with reasons, date(s) of occurrence, and actions taken. All the deviation should be notified to respective ethics committee.

7.5. Audits and inspections

Any aspect of the clinical trial may be subject to audits conducted by Sponsor Quality assurance team or inspections from national or international regulatory authorities or from IRBs/IECs. The purpose of a sponsor's audit, which is independent of and separate from routine monitoring or quality control functions, is to evaluate trial conduct and compliance with the protocol, SOPs, GCP, and the applicable regulatory requirements.

8. STUDY TREATMENT

8.1. Study Drug

Test drug:

Elobixibat tablet (10 mg oral once daily self-administered as two tablets of 5 mg) before Breakfast from Day 1.

Comparator:

Matching placebo (10 mg) self-administered oral once daily as two tablets before breakfast from Day 1.

8.2. Administration

- The usual adult dosage is 10 mg of elobixibat self-administered orally once daily before breakfast from day 1. The dose may be adjusted according to the patient's symptoms, but the maximum daily dose will be 15 mg.
- Since abdominal pain and diarrhea may occur during administration of this drug, it is necessary to consider reducing the dose, suspending or discontinuing the drug depending on the symptoms, and to continue administration of this drug on a regular basis so as not to continue administration of this drug indiscriminately.

8.3. Storage Requirements

All study drug supplies will be stored as per COA or as per sponsor’s / manufacturer’s instructions. Until dispensed to the subjects, the study drug will be stored in a secure, temperature controlled, restricted access area accessible to authorized personnel only

8.4. Concomitant treatment

- Prohibited Medication:

Prescription and over-the-counter (OTC) laxatives other than those prescribed as a rescue medication during the baseline/washout period by the Investigator.

Note: the use of fiber, bulk laxatives, stool softeners [surfactants such as docusate], and probiotics are acceptable, provided that the patient has been on a stable dose for 30 days before the Screening Visit and plans to continue stable dosing for the duration of the trial.

- Significant change in diet.
- Over-the-counter or prescription laxatives cannot be administered during the pre-treatment period(s).

All medicine listed below ("1-day Washout" and "14-day Washout") are excluded during the Pretreatment and Treatment Periods.

- A 1-day washout means that the particular medicine is not allowed during the calendar day before the Pretreatment Visit
- A 14-day washout means that the particular medicine is not allowed during the 14 calendar days before the Pretreatment Visit

1-Day Washout (no medicine during the calendar day before the Pretreatment Visit)

1. Any over-the-counter or prescription laxative, suppository, or enema (e.g., polyethylene glycol, lactulose, Fleet's) and any herbal or natural agent that a person might take for constipation. Note: The use of fiber, bulk laxatives, stool softeners (surfactants such as docusate), and probiotics is acceptable, provided the patient has been on a stable dose during the 30 days before the Screening Visit and plans to continue stable dosing throughout the trial.
2. Any medicine used to treat diarrhea (e.g., bismuth subsalicylate, kaolin)
3. NSAIDs if taken for abdominal pain or discomfort.
4. Others: Midazolam, Aluminum containing antacid.

14-Day Washout (no medicine during the 14 calendar days before the Pretreatment Visit)

1. Drugs with known pharmacological activity at 5-HT4, 5-HT2b or 5-HT3 receptors (e.g., cisapride, tegaserod, ondansetron, tropisetron, granisetron, dolasetron, and mirtazapine).
2. Any treatment specifically taken for IBS-C or CIC alone or in combination, including lubiprostone, an approved chloride channel activator that enhances intestinal fluid secretion, linaclotide, plecanatide, colchicine, and misoprostol. Note: patient has taken commercially available elobixibat, linaclotide or participated in a linaclotide or plecanatide or elobixibat clinical study during the 30 days before the Screening Visit.
3. Prokinetic agents (e.g., metoclopramide, itopride, prucalopride, and domperidone).
4. Anti-cholinergic agents (e.g., dicyclomine, flavoxate, scopolamine, hyoscyamine, propantheline, oxybutynin, tolterodine, solefenacin, darifenacin, and trospium). Note: inhaled ipratropium and tiotropium are permitted.
5. Bile acid sequestrants (e.g., cholestyramine and colestipol).

6. Cholinomimetic agents (e.g., bethanechol, pyridostigmine, tacrine, and physostigmine). Note: intraocular cholinomimetic agents (e.g., pilocarpine) are permitted.
7. Antipsychotic agents (e.g., risperidone, haloperidol, droperidol, chlorpromazine, perphenazine, all phenothiazines, quetiapine, olanzapine, and clozapine) unless the patient has been on a stable dose for 30 days before the Screening Visit and there is no plan to change the dose after the Screening Visit. Note: paliperidone is permitted without restriction.
8. Antidepressants unless the patient has been on a stable dose for 30 days before the Screening Visit and there is no plan to change the dose after the Screening Visit. Specifically included are the following: - Tricyclic antidepressants (e.g., amitriptyline, imipramine, and nortriptyline); - Monoamine oxidase inhibitors (e.g., furazolidone, isocarboxazid, pargyline, phenelzine, and selegiline tranylcypromine); - Selective serotonin reuptake inhibitors (e.g., fluoxetine, sertraline, paroxetine, and citalopram); - Serotonin and norepinephrine reuptake inhibitors (e.g., venlafaxine and desvenlafaxine succinate) - Others (e.g., trazodone, and bupropion).
9. Calcium channel blocker verapamil unless the patient has been on a stable dose for 30 days before the Screening Visit and there is no plan to change the dose after the Screening Visit. Note: all other calcium channel blockers (e.g., nifedipine, diltiazem, amlodipine, felodipine, nicardipine, nimodipine, nisoldipine, etc.) are permitted and may be used without restriction.
10. Oral and parenteral antibiotics (However, a standard regimen [up to 10 days] of oral antibiotics is permitted.).
11. Any investigational or imported drugs that have not been approved for human use by the US FDA.
12. All narcotics either alone or in combination (e.g., tramadol, codeine, morphine, propoxyphene, loperamide, diphenoxylate, and paregoric). Note: narcotics used as anesthesia for a colonoscopy require a 5 calendar day wash-out prior to the patient entering into the Pretreatment Period.
13. Any medicine taken for the purpose of losing weight (e.g., orlistat, phentermine, phendimetrazine, diethylpropion, benzphetamine, and sibutramine).
14. Any medicine that is known to cause diarrhea (e.g., acarbose).

15. Proton pump inhibitors (e.g., omeprazole, lansoprazole, esomeprazole, pantoprazole, rabeprazole) unless the patient has been on a stable dose for 30 days before the Screening Visit and there is no plan to change the dose after the Screening Visit.
16. Others: barbiturates (e.g., butalbital and phenobarbital) and chronic oral or parenteral glucocorticoids (which must be discontinued at least three months before screening; however, one 10-day course of oral or 1 injection of parenteral glucocorticoids is permitted), Ursodeoxycholic acid, chenodeoxycholic acid, Sucralfate hydrate, aldioxa, etc. Cholestyramine, Digoxin, dabigatran etexilate, and methane sulphonate. Pregabalin, is acceptable, provided the patient has been on a stable dose during the 30 days before the Screening Visit and plans to continue stable dosing throughout the trial.

- Permitted concomitant medications:

Concomitant medicines are defined as any medicines taken during the treatment period (i.e., between the date of the first dose of study drug in the treatment period and the date of the last dose of study drug in the treatment period, inclusive). Any medicines started after the date of last dose of study drug will not be considered concomitant medicines. Both prior and concomitant medicine use will be summarized by the number and proportion of patients in each treatment group receiving each medicine within each therapeutic class. Concomitant medicine will be summarized for the treatment period using the Safety Population.

9. ADVERSE EVENTS

9.1. Adverse Events (clinical)

At each assessment, all adverse events, whether previously known or not, will be recorded with their description, intensity (severity), action taken, duration, outcome and opinion about causal relationship to the study drugs. In case of any specific Medical events of Special interest, these shall be mentioned with all required details.

9.2. Adverse Events (laboratory and others)

Any clinically significant change in any of the lab parameters evaluated at the end of the study or during the study conduct would be recorded as an investigational adverse event.

9.3. Collection of Adverse Events

It is the responsibility of the Investigator to collect all AEs (both serious and non-serious) derived by spontaneous, unsolicited reports of subjects, by observation and by routine open questionings e.g., "How has you felt since I last saw you?"

9.4. Definitions

An AE is any untoward medical occurrence that occurs in a subject or clinical investigation subject administered a pharmaceutical product, and which does not necessarily have to have a causal relationship with this treatment. An AE can therefore be any unfavorable and unintended sign (including abnormal laboratory finding), symptom, or disease temporally associated with the use of an investigational product, whether or not considered related to the product.

All AEs, including concurrent illnesses, occurring during the study will be documented in the subject's Case Report Form (CRF). Concomitant illnesses, which existed before entry into the study, will not be considered AEs unless they worsen during the treatment period. All AEs, regardless of the source of identification (e.g., physical examination, laboratory assessment), must be documented.

Pre-existing conditions will be recorded in the subject's Screening Case Report Form (CRF) on the Medical History page.

A treatment-emergent AE (TEAE) will be defined as an AE that begins or that worsens in severity after at least one dose of study drug has been administered.

9.5. Assessment of Adverse Events

Each AE will be assessed by the Investigator with regard to the following categories.

9.5.1. Seriousness

A 'serious' adverse event is one that is fatal or life threatening, lead to permanent disability, or requires hospitalization.

Serious events fall under the criteria as below:

- Results in death
- Is life-threatening*
- Requires inpatient hospitalization or prolongation of existing hospitalization

- Results in persistent or significant disability/incapacity or
- Causes a congenital anomaly/birth defect
- Medically significant events (or) Important Medical events (IME)

*A `life-threatening' adverse event places the subject at immediate risk of death in the judgment of the investigator.

Important Medical Events are considered to be of significant medical magnitude (based on medical judgment) such that the event may jeopardize the subject to an extent that it may require medical or surgical intervention to prevent one of the outcomes listed as an SAE. Examples of such medical events include, allergic bronchospasm requiring intensive treatment in an emergency room or at home, blood dyscrasias or convulsions that do not result in hospitalization, or development of drug dependency or drug abuse.

The seriousness criterion of "medically significant" should only be selected when none of the other seriousness criteria apply to the event but the Investigator still considers the event as serious.

An `unexpected' adverse event is one not identified in nature, severity, or frequency in the investigator's brochure or the product package insert for the study drug.

9.5.2. Intensity (severity)

The intensity of each AE must be assessed by the Investigator using one of the following categories and recorded in the subject's Case Report Form (CRF).

Description of Intensity (severity) of Adverse events

The NCI Common Terminology Criteria for Adverse Events version 5.0 (Current version) is a descriptive terminology which can be utilized for Adverse Event (AE) reporting. A grading (severity) scale is provided for each AE term.

Adverse Event Terms

An AE is any unfavorable and unintended sign (including an abnormal laboratory finding), symptom, or disease temporally associated with the use of a medical treatment or procedure that may or may not be considered related to the medical treatment or

procedure. An AE is a term that is a unique representation of a specific event used for medical documentation and scientific analyses.

Serious Suspected Unexpected Adverse Reaction (SUSAR)

An unexpected adverse reaction (UAR) is an adverse reaction that is not consistent with the product information in the IB/SmPC. A suspected unexpected serious adverse reaction (SUSAR) is any UAR that at any dose:

- Results in death;
- Is life threatening (i.e. the subject was at risk of death at the time of the event; it does not refer to an event which hypothetically might have caused death if it were more severe);
- Requires hospitalisation or prolongation of existing hospitalisation;
- Results in persistent or significant disability or incapacity;
- Is a congenital anomaly or birth defect.
- Medically significant

Adverse Drug Reaction (ADR)

An ADR is any untoward and unintended response to an IMP related to any dose administered. The definition implies a reasonable possibility of a causal relationship between the AE and the IMP. This means that there are facts (evidence) or arguments to suggest a causal relationship, the relationship cannot be ruled out.

Medical Event:

A medical event is defined as any untoward medical occurrence in a patient or a healthy volunteer after signing the ICF till the administration of the first dose of the investigational medicinal product.

An SAE which occurs before administration of study drug (e.g. during screening) will not be considered for expedited reporting to the authorities.

An SAE which occurs as a result of any screening procedure will also not be considered for expedited reporting to the authorities but will be entered in the subject's source records and the CRF.

Grades of Severity

Grade refers to the severity of the AE. In oncology studies as an example, The CTCAE v 5.0 displays Grades 1 through 5 with unique clinical descriptions of severity for each AE based on this general guideline:

- Grade 1 Mild AE
- Grade 2 Moderate AE
- Grade 3 Severe AE
- Grade 4 Life-threatening or disabling AE
- Grade 5 Death related to AE

9.5.3. Causality assessment

The Investigator will assess the causality / relationship between the study drug and the AE and record that assessment in the subject's enrollment Case Report Form (CRF).

Description of causal relationship of adverse events to the study drug regimen:

Definite: The event occurred with a plausible time relationship to medicine use; it could not be explained by concurrent diseases, or other medicines or substances; Withdrawal of the medicine resulted in recovery or significant improvement, and re-initiation ("re-challenge") of the medicine, if it was appropriate, resulted in recurrence of the same event; and the event was a recognizable pharmacological action of the medicine or a clearly identifiable clinical event.

Probable: means the occurrence of the event was temporally associated with administration of the medicine, it was unlikely to be caused by a concurrent disease or other medicine or substance, and withdrawal of the medicine resulted in improvement or recovery.

Possible: means the occurrence of the event was temporally associated with administration of the medicine, but it could also be explained by concurrent disease or other medicine or substance, and/or details such as administration dates or response to withdrawal of the medicine may have been absent or unclear.

Unlikely: means the event occurred at a time in relation to medicine administration, which made causation improbable. Concurrent disease or other medicine or substance may have provided a plausible explanation.

Unclassified: means more data were required in order to assess the adverse event.

Unclassifiable: means the data available were insufficient or contradictory, and it was not possible to obtain further data or verify existing data.

9.6. Recording of Adverse Events

Adverse event reporting will extend from signing of informed consent until Visit 5/Early Termination visit. All AEs, regardless of the relationship to study drug, will be recorded in the subject's Case Report Form (CRF).

The Investigator will record all AEs, regardless of relationship to study drug on the adverse event page in the in the subject's Case Report Form (CRF). Standard medical terminology should be used when describing AEs. Whenever possible a diagnosis should be made and recorded on the CRF rather than listing signs and symptoms. Intermittent AEs can be recorded once. The anatomical location of the AEs must be specified where applicable.

The following information should be recorded in the subject's Case Report Form (CRF).

<u>Description:</u>

- Severity of the event (see Intensity for details)
- Study drug use continued or not
- Outcome of the event (resolved, persistent, unknown, death)
- Relationship to study drug (see Relationship to Study drug (Causality) for details)
- Indication of whether the event is serious (see Seriousness for details)
- Actions taken including treatment with concomitant medication

9.6.1. Procedures for Reporting of Adverse Events

All serious adverse event (SAE) that occur during the study period, whether or not considered to be related to the study drug, must be reported (typed in English) to the Sponsor, within 24 hours of the information becoming available.

These SAE reports must contain the following information:

A. Study name/number

B. Study Drug

C. Investigator details (name, phone, fax, e-mail)

D. Subject Number

E. Subject Initials

F. Subject Demographics

G. Clinical Event

1) Description

2) Date of onset

3) Treatment (drug, dose, dosage form)

4) AE Relationship to study drug

5) Action taken regarding study drug in direct relationship to the AE

6) Results of tests or investigations, which are relevant for interpretation of SAE

7) Outcome of the event (Recovered/ recovered with sequelae, Not recovered, Recovering)

H. If the AE was fatal

1) Cause of death (whether or not the death was related to study drug)

2) Autopsy findings (if available)

The SAE form completion and reporting must not be delayed even if all of the information is not available at the time of the initial contact. Additional information (follow-up) about any SAE unavailable at the initial reporting should be forwarded within 24 hours of the information becoming available. Subjects who have had an SAE during the treatment period must be followed clinically until all parameters (including laboratory) have either returned to normal or have stabilized or are otherwise explained.

9.7. Reporting of Serious Adverse Events

The CDSCO, in pursuance to implementation of the e-Governance has launched various online services through the portal "SUGAM" (www.cdscoonline.gov.in) on 14.11.2015. The stakeholders were requested to avail this facility which is intended to further reduce

the time and transaction cost. Therefore, it is effective from 14.03.2021, and from this date online files of SAE reports were to be sent for processing through SUGAM portal. All SAEs should be reported to Central Licensing Authority via SUAGM portal.

All SAEs should be reported by the investigator to the Licensing authority (via SUGAM portal), Sponsor and to the Ethics Committee that accorded approval to the study protocol within 24 hours of their occurrence/awareness

Investigator must notify all the SAEs to the sponsor representatives listed below via email or fax:

Sponsor contact:

Tel: +91-40-49002429

Email ID: pharmacovigilance@drreddys.com

AND

nitus@drreddys.com

If the outcome of the SAE is fatal i.e. SAE results in death, the report on SAE of death after due analysis (Table 5 of schedule III of New Drugs and Clinical Trials Rules 2019

G.S.R. 227(E)) shall be submitted by the investigator to the DCGI (Central Licensing Authority) via SUGAM portal, the Chairman of the ethics committee that accorded permission to conduct the study, and to the Head of the institution where the trial has been conducted, within 14 calendar days of knowledge of occurrence of the SAE.

The Central Licensing Authority should forward the report of the Investigator, Sponsor or its representative (whosoever had obtained permission from the Central Licensing Authority for conducting clinical trial) and the Ethics Committee to the Chairperson of the Expert Committee.

If the SAE is permanent disability or any other injury other than death, the reports (Table 5 of schedule III of New Drugs and Clinical Trials Rules 2019 G.S.R. 227(E)) on SAE, after due analysis should be submitted by the investigator to the DCGI (Central Licensing authority) via SUGAM portal, the chairperson of ethics committee and to

Head of the institution where the trial has been conducted, within 14 calendar days (or as per applicable regulatory timelines) of reporting of the event.

In case of a serious adverse event of death, the sponsor or its representative shall forward the reports on SAE after due analysis to the DCGI (Central Licensing authority) via SUGAM portal, Chairperson of the Ethics committee, and Head of the institution, where the trial has been conducted within 14 calendar days of knowledge of occurrence of the SAE.

In case of any SAE of permanent disability or any other injury other than deaths, the sponsor or its representative shall forward the reports on SAE after due analysis to the DCGI (Central Licensing authority) via SUGAM portal, Chairperson of the Ethics committee, and Head of the institution, where the trial has been conducted within 14 calendar days of reporting of SAE.

All SAE reports shall be submitted to the DCGI as per – table 5 of schedule III of New Drugs and Clinical Trials Rules 2019 G.S.R. 227(E), in accordance with the format and checklist (based on the event). The sponsor will submit all SAE report to CDSCO via SUGAM portal as per table 5 of schedule III of New Drugs and Clinical Trials Rules 2019 G.S.R. 227(E). The sponsor or its representative shall submit all SAE reports to CDSCO as per table 5 of schedule III of New Drugs and Clinical Trials Rules 2019 G.S.R. 227(E). The SAE reports will be submitted with proper binding, indexing and page number.

Sponsor shall share the due analysis report with all other participating Investigator(s) / study site team(s) and same has to be notified by site team(s) to their respective IECs

All adverse events, independent of relationship to study drug, must be reported starting from time of first administration of study drug until end of study or early termination. Subjects will be followed-up for adverse events until complete resolution.
All SAEs will be followed until resolution, stabilisation, until the event is otherwise explained, or the patient is lost to follow-up.

A event which occurs before administration of study drug/procedure (e.g. during screening/as a result of screening, after signing the ICF and before administration of the study drug, during check-in/after check-in and before administration of the study drug)

will not be considered as adverse event for the investigational product and hence it will not be reportable as adverse event for the investigational product to the applicable regulatory authorities (e.g. DCGI, etc.).

If there is any change in the Pharmacovigilance regulation by the DCGI (licensing authority) in regard to the SAE reporting, the timelines for SAE reporting, the concerned authorities/ personnel to whom the SAE's has to be reported then the applicable Pharmacovigilance regulations will be followed by Investigator / Sponsor. The investigator / Sponsor will comply to the changes in the Pharmacovigilance regulations (SAE reporting and timelines for SAE reporting), by the DCGI (licensing authority) on their responsibilities. The investigator will cooperate with the sponsor in complying to the change in the Pharmacovigilance regulation (SAE reporting and timelines for SAE reporting), as applicable.

Serious adverse event (SAE) occurring during a clinical trial should be communicated promptly (within 14 calendar days) by the Sponsor or its representative to the other Investigator(s) participating in the study.

Investigator Reporting of Pregnancy - Notifying the Study Sponsor

All patients who participate in the study should be counselled on the need to practice adequate birth control and on the importance of avoiding pregnancy during study participation as detailed in the inclusion and exclusion criteria. Serum pregnancy testing will be performed at screening. A subject who is found to be pregnant at the screening visit will be excluded from the study and will be a screening failure. Patients who have been enrolled in the study should be instructed to contact the Investigator or study staff immediately if pregnancy occurs or is suspected. Early termination visit assessments are required as soon as possible after learning of the pregnancy. Pregnant females will be discontinued from study treatment by the Investigator. A male that has a partner that becomes pregnant during the study will not be discontinued from study treatment, however the pregnancy will be reported and documented. Urine pregnancy will be performed at subsequent visits, if applicable.

Details of the pregnancy should be recorded on the Pregnancy Report form and reported Dr. Reddy's Laboratories Limited within 24 hours of awareness by email

pharmacovigilance@drreddys.com from the time of initial awareness, even if beyond the closure of the clinical database.

9.7.1 Compensation in case of injury or death during clinical trial

In case of an injury occurs to any subject during clinical trial, the sponsor, shall provide the cost for medical management to such subject as long as required as per the opinion of investigator or till such time it is established that the injury is not related to the clinical trial, as the case may be, whichever is earlier.

In case of clinical trial related death or permanent disability or any other injury other than deaths, the sponsor or its representative shall pay the quantum of compensation as decided by the Central Licensing Authority, within thirty days of the receipt of such order.

In the event of an injury, not being permanent nature, the quantum of compensation shall be commensurate with the loss of wages of the subject as provided in the seventh schedule of The New Drugs and Clinical Trials Rules 2019 G.S.R. 227(E).

In case of permanent disability or any other injury occurs to any subject during clinical trial, the trial subject shall be provided financial compensation by the sponsor or its representative, as per order of the Licensing Authority defined under sub rule 3 of rule 42 of chapter VI of New Drugs and Clinical Trials Rules 2019 G.S.R. 227(E), and the financial compensation shall be in addition to any expenses incurred on the medical management of the trial subject.

In the case of death occurs to the trial subject during clinical, his/her nominee(s) should be entitled for financial compensation by the sponsor or its representative, as per the order of the Central Licensing Authority defined under sub rule 2 of rule 42 of chapter VI of New Drugs and Clinical Trials Rules 2019 G.S.R. 227(E), and the financial compensation shall be in addition to any expenses incurred on the medical management of the trial subject

Known Adverse Events with the study drug

In CIC clinical trials, the most common adverse reactions in LINZESS-treated patients (incidence ≥2% and greater than placebo) were (i) diarrhea (16% vs 5% placebo), (ii) abdominal pain (7% vs 6%), (iii) flatulence (6% vs 5%), (iv) upper respiratory tract

infection (5% vs 4%), (v) sinusitis (3% vs 2%) and (vi) abdominal distension (3% vs 2%).

10. ETHICAL CONSIDERATIONS

All ethical and regulatory practices should be adopted and practiced throughout the trial by all the concerned trial personnel, as outlined in the ICH-GCP guidelines and applicable regulatory requirements.

10.1. Ethics Committee review and communications

Study documents (protocol, PIS/ICF, etc…) will be submitted by the investigator to the respective ethics committee for its review and approval, after receipt of approval letter from ethics committee along with other local regulatory approval, the study will be initiated at the respective sites.

10.2. Informed Consent Process

Informed Consent is documented by means of a **written, signed and dated** informed consent form.

According to ICH GCP guidelines 1.28 Informed consent process defined as *"Process by which a subject **voluntarily** confirms his / her willingness to Participate in a particular trial, after having been informed of all aspects of the Trial that are relevant to the Subject's decision to participate".*

10.2.1. LAR (Legally Acceptable Representative)

“An individual or juridical or other body authorized under applicable law to consent, on behalf of a prospective subject, to the subject's participation in the clinical trial.

10.2.2. Impartial Witness

A person, who is independent of the trial, who cannot be unfairly influenced by people involved with the trial, who attends the informed consent process if the subject or the subject’s legally acceptable representative cannot read, and who reads the informed consent form and any other written information supplied to the subject.

10.2.3. Re-consenting Process

Re-consenting may be required for various reasons including but not limited to cases where:

- When significant new information comes to light that affects the safety of the subjects and/or might have influenced the subject’s original decision to take part.
- Protocol Amendments may require the ICF to be revised
  E.g. Changes in study procedures, entry criteria, patient numbers etc
  Sponsor decides whether the ICF needs to be modified

10.3. Subject Confidentiality

- Subject's identity will be maintained confidential and confidentiality of records and documents that could identify subjects will be protected, respecting the privacy and confidentiality rules in accordance with applicable regulatory requirements.
- Subjects must be identified only by their assigned identification number and initial on all CRFs and other records and documents submitted to sponsor.
- The investigator will keep a Patient Identification List with complete identification information (name, address, contact number etc...) on each subject.
- Documents not for submission to sponsor such as subject's written informed consent form should be maintained by the investigator in strict confidence.
- Monitors and auditors from sponsor, and representatives of IEC or other regulatory agencies will be granted direct access to subject medical records and other study documents for verification of study procedures and data without violating the confidentiality of the subject.
- The subject should be informed that by signing a written informed consent form, the subject or his/parent or guardian is authorizing such access.

11. STUDY MONITORING

11.1. Monitoring Frequency and Agenda

Monitoring will be conducted based on the study phase, complexity and duration etc. and relevant information will be shared with the Investigators and staff regarding monitoring in advance. During each visit the monitor will discuss with the Investigator and Coordinator all relevant issues pertaining to the trial, validate the CRFs with the source data, identify and request for clarification in case of any discrepancies or errors. The Investigator / delegated site staff will provide source documents, completed CRFs, space and time for resolving the queries during the monitoring visit. The monitor will collect completed CRFs during each visit.

11.2. Source Data Verification

The source data verification and review of records is necessary to minimize any errors in transcribing data onto the CRFs. The monitor will validate the CRF entries with the source data and subjects' records.

11.3. Data Clarification / Rectification

Data will be entered into the CRF by site personnel. Post data entry, source data verification will be completed by Study monitor / Clinical Research Associates (CRA). If any query requires clarification from site, data management personnel will raise the query. Based on query raised by data management personnel, site will respond to the query. If response for raised query is satisfactory, then data management personnel will close the query or if response for raised query is not satisfactory then data management personnel will re- query to site.

11.4. Close-out Meeting / Site Closure

At this visit, left over trial medication(s) and trial related materials will be collected. An accounting will be done of all materials supplied to the site, as well as all subjects screened and enrolled.

11.5. Trial Supplies Accounting & drug dispensing log

The trial medication dispensed /consumed must correspond with that sent to the site, as the use of this medication is restricted only for trial purposes. On completion of the trial, all remaining medication must be handed over to the sponsor.

11.6. Records Preservation and Archiving

The investigator & the sponsor will have to archive all documents related to the trial at their respective sites for a period of 05 years and /or as per the sponsor's decision. In the event of the investigator leaving the institution, the same must be handed over to his successor who will maintain custody of the documents. The information of the same should be sent to the sponsor.

11.7. Subject Screening Log

The trial site will maintain a record of all patients screened. This log will help to evaluate the total percentage of patients found eligible, from among those screened. Patient Enrolment and Visit Log

The trial site will maintain a log of all patients enrolled into the study in order that each patient entering into the study is accounted for. This is necessary to identify those subjects who are not on schedule for their scheduled visits and may need to be reminded to conform to follow up schedules.

11.8. Patient Identification Log

A log of patient identification will be maintained at the trial site. This is necessary to contact subjects to conform to follow up schedules or licensing authority auditing purposes.

11.9. Informed Consent Forms

The informed consent is a process by which a subject voluntarily confirms his or her willingness to participate in a particular trial, after having been informed of all aspects of the trial relevant to the subject's decision to participate. The informed consent sheet will be provided in the regional language along with the English copy. Prior to screening each patient must give his consent for study participation in writing. The informed consent must be obtained in duplicate. One copy will be retained by the investigator & the other copy is to be given to the patient. If required, Audio-visual (AV) recording of entire process of subjects' Informed consent will be conducted in clinical trials, as per site specific or regulatory requirements.

11.10. Source Documents

These comprise the patient's hospital case sheets; laboratory investigation reports, ECGs etc. These will be used to transcribe data onto the CRFs. These will have to be

made available to the sponsor's representative at the time of monitoring in order to validate CRF entries.

11.11. Study Documents

The sponsor will supply copies of protocol, patient informed consent form/patient informed assent form and patient information sheet [in English and relevant vernacular languages], diaries to the investigator. The sponsor will ensure that the translations and back- translations of the patient informed consent form/patient informed assent form and patient information sheet from English to the relevant vernacular languages is done in an appropriate manner and take certificate of translation from the translating agency.

12. INVESTIGATIONAL PRODUCT MANAGEMENT

12.1. Study Drugs

Test drug: Elobixibat 5 mg

Comparator: Placebo matching Elobixibat 5 mg tablet

The sponsor will supply the study drugs to the investigators duly labeled and packed. The sponsor will supply rescue medicine but not the concomitant medication.

Method of assigning treatment: Eligible subjects after confirmation of diagnosis and conformance with entry criteria will be randomized to any of the treatment groups. Study Medication will be dispensed to the randomized subjects.

Storage conditions: All study drug supplies will be stored as per COA or as per sponsor's / manufacturer's instructions. Until dispensed to the subjects, the study drug will be stored in a secure, temperature controlled, restricted access area accessible to authorized personnel only.

12.2. Packaging, Labeling and Storage

All study drug supplies will be labeled and must be stored in accordance with the sponsor's / manufacturer's instructions. Until dispensed to the subjects, the study drug will be stored in a securely locked area, accessible to authorized personnel only.

12.3. Unused investigational products

The unused investigational products will be accounted for at all the sites at close out visit and must be handed over to the sponsor.

12.4. Blinding and Unblinding Procedures

Blinding is a procedure in which one or more parties to the trial are kept unaware of the treatment assignment(s). Single-blinding usually refers to the subject(s) being unaware, and double-blinding usually refers to the subject(s), investigator(s), monitor, and, in some cases, data analyst(s) being unaware of the treatment assignment(s).

12.4.1. Randomization

The randomization codes will be generated by the Randomization Code Administrator, an individual within Statistical Programming who will generate the randomization codes and will not be assigned the role of Statistical Programmer for this study. The randomization codes will be provided in a secure manner.

12.4.2. Unblinding

Unblinding sealed envelopes will be provided to the site. In case the unblinding becomes mandatory, the investigator has to open the envelope carrying the randomization code of the subject and find out the study medication being given to the subject. Utmost care should be taken to ensure that the person unblinding the randomization code opens the correct envelope for the particular subject who requires unblinding.

If the blind is broken, the date, time, and reason must be recorded in the subject’s source record, CRF, and any associated AE report. In case of accidental unblinding, the unblinding incident and unblinded subject must be listed as a major protocol deviation. Persons going for monitoring the study should ensure that unblinding (accidental / emergency) has not occurred at the site and blinding has been completely ensured. In case an unblinding has happened, the monitor should document the circumstances and reasons for unblinding.

.

12.4.2.1. Accidental Unblinding

In case of an accidental unblinding, the investigator shall promptly document and explain in detail to the sponsor the reasons for / situation that led to unblinding and inform the responsible Ethics committee. However, it is the responsibility of the investigator to ensure that accidental unblinding does not occur.

12.4.2.2. Unblinding for Regulatory and Analysis requirement at the end of study

For regulatory requirement, reporting of SAEs not previously unblinded at the site level, the blind will be broken for SAEs, in case of requirement.

The overall randomization code will be broken only when all final clinical data have been entered onto the database and all data queries have been resolved, and the assignment of subjects to the analysis sets has been completed.

12.5. Supplies Accountability

As the above is an investigational study, the trial medication is to be used for trial purposes only. For this reason, a log has to be maintained of all study medication received, consumed, left over. The remaining medication will be collected by the sponsor's representative at the time of trial closure. The remaining stationary will also be collected by the sponsor's representative at the time of trial closure and kept at the sponsor's place.

13. STATISTICS and DATA ANALYSIS

13.1. Study Subjects

Male and non-pregnant female subjects (18-65 years of age) with clinical diagnosis of chronic constipation will be included in the trial.

13.2. Sample Size Estimation

A total of 150 patients with chronic constipation will be enrolled in the study.
Assuming a significance level of 2.5% and a power of 95%, we require 60 completed subjects per treatment group (a total of 120 subjects in study) to prove superiority of Elobixibat.

Assuming ~20% drop-out rates, 150 subjects will be randomized in this study with 1:1 ratio (75 subjects per each arm) to Elobixibat 5mg vs Placebo.

13.3. Statistical Analysis

A Statistical Analysis Plan (SAP) will be prepared as a separate document and finalized before database lock. Any deviation from the original statistical plan will be described and justified in the final report, as appropriate. The procedure for accounting for missing, unused and spurious data will be included in the Statistical Analysis Plan. All statistical analysis will be conducted using SAS®, Version 9.4 or higher.

The accepted per-protocol (PP) population used for evaluation includes all randomized subjects who meet all inclusion/exclusion criteria, dosed a pre-specified proportion of the scheduled doses of the assigned product for the specified duration of the study, do not miss a pre-specified number of scheduled doses for more than pre-specified number of days, and complete the evaluation within the designated visit window with no protocol violations that would affect the treatment evaluation. The protocol should specify how compliance will be verified, (e.g., by the use of subject diaries).

The mITT and safety populations include all randomized subjects who use at least one dose of product.

Subjects who are discontinued early from the study due to lack of treatment effect should be included in the PP population. Subjects whose condition worsens and who require alternate or supplemental therapy, excluding pre-specified rescue therapies, for the treatment of constipation during the treatment phase of the study should be discontinued, included in the mITT and PP population analysis, and provided with effective treatment. Subjects discontinued early for other reasons should be excluded from the PP population but included in the mITT population. The protocol should clearly state how missing data will be handled in the statistical analyses and provide appropriate justification for the method chosen.

13.3.1. Efficacy analysis

The primary efficacy parameter is Change from baseline in weekly frequency of Spontaneous Bowel Movement (SBMs).

The primary efficacy analysis the proportion of 2-week SBM Overall Responders will be compared to the proportion in the placebo group using the CMH test.

The secondary efficacy parameters are:

1. Proportion of patients with complete spontaneous bowel movement (CSBM)
2. Proportion of patients with a SBM within 24 h after the first dose of study drug
3. Median time to first SBM
4. Change from baseline in Stool consistency using Bristol Stool Form Scale (BSFS)
5. Change from Baseline in Weekly Degree of Straining of SBMs
6. Change from Baseline in Weekly Abdominal Bloating Score
7. Change from Baseline in Weekly Abdominal Discomfort Score

For each of the above secondary efficacy parameters, Elobixibat dose group will be compared to the placebo group using an analysis of covariance (ANCOVA) model with fixed effect terms for treatment group and the patient's corresponding baseline value of the parameter as a covariate. The overall type I family-wise error rate for testing the primary and secondary efficacy parameters will be controlled at the 0.05 significance level using the following five-step serial gatekeeping multiple comparisons procedure (MCP).

All hypothesis tests will be two-sided.

The first step will test the primary efficacy parameter for the Elobixibat 5mg dose group at the 0.05 significance level

The second step will test the primary efficacy parameter for the 5mg Elobixibat dose group and the first five secondary parameters (i.e., SBM, CSBM, stool consistency, severity of straining, and constipation severity).

The third step will test the last two secondary parameters (i.e., bloating, abdominal discomfort) for the Elobixibat 5mg dose group. The two individual hypotheses within this step will be tested using an overall type I error rate of 0.05 by means of a Hochberg procedure to control for multiple parameters.

13.3.2. Safety Analysis

The safety analysis will be performed using the Safety Population. The safety parameters include AEs, clinical laboratory parameters, vital signs, and ECG parameters. For each safety parameter, the last assessment made before randomization will be used as the baseline for all analyses of that safety parameter.

13.4. Data handling

All clinical data generated during the conduct of the study will be entered in the source notes and will be transcribed in the respective case record form (CRF). The computer-

generated randomization schedule will also be treated as raw data. All raw data and transcribed data forms compiled by the study personnel assisting in the study will be checked for completeness. All data related to the project will be in the custody of the Principal Investigator or Project Manager until transferred to archives.

The detail regarding CRF completion requirements, correction requirements, including who is authorized to make corrections on the CRF and how queries about study data are handled will be available in data management plan. All raw data generated during the conduct of the project compiled by the study personnel assisting in the study will be checked for completeness. Biostatistics department after receipt of the raw data will perform a statistical analysis and statistical data will be generated which will be further sent for compilation of the final clinical study report of the study.

14. QUALITY CONTROL AND QUALITY ASSURANCE

Quality control and quality assurance process will be followed as per relevant in-house standard operating procedures.

15. INVESTIGATOR RESPONSIBILITIES AND ESSENTIAL DOCUMENTS IN STUDY

The investigator is accountable for the conduct of the trial at his/her site and must ensure adequate supervision of the conduct of the trial at the trial site. If any tasks are delegated, the investigator must maintain a log of appropriately qualified persons to whom he/she has delegated specified trial-related duties. The investigator must ensure that there is adequate and documented training for all staff participating in the conduct of the trial. It is the investigator's responsibility to supervise the conduct of the trial and to protect the rights, safety, and well-being of the subjects. A qualified physician, who is an investigator or a sub-investigator for the trial, must be responsible for all trial-related medical decisions.

The investigator is responsible for filing essential documents (i.e. those documents which individually and collectively permit evaluation of the conduct of a trial and the quality of the data produced) in the investigator trial master file. The documents including the subject identification or code list must be kept in a secure locked facility, so no unauthorized persons can get access to the data. The investigator will take all necessary safety measures to prevent accidental or wrongful destruction, loss or deterioration of data. The investigator will prevent any unauthorized access to data. During any period of unavailability, the investigator must delegate responsibility for

medical care of subjects to a specific qualified physician who will be readily available to subjects during that time.

If the investigator is no longer able to fulfil the role as investigator (e.g. if he/she moves or retires), a new investigator, who is suitably qualified and trained, will be appointed in place.

16. FINAL STUDY REPORT

A final report will be prepared, integrating medical and statistical aspects. The Investigators will be provided with a copy of the summary of the final report, where requested.

16.1. Publication Policy

The authorship will include sponsor representatives who were involved in the design and conduct of this study, and investigators authorship will be based on enrollment status attained by each investigator, since enrollment is on a competitive basis. Authors will be selected based on the authorship criteria mentioned by International committee of medical journal editors.

Sponsor has the final rights to decide the authorship as per sponsor publication policy.

DocuSign Envelope ID: DCBE1147-372F-4B2F-B3C1-63958E711F1B

17. REFERENCES

1. Andrews CN, Storr M. The pathophysiology of chronic constipation. Can J Gastroenterol. 2011;25 Suppl B(Suppl B):16B-21B.
2. Dennison C, Prasad M, Lloyd A, Bhattacharyya SK, Dhawan R, Coyne K. The health-related quality of life and economic burden of constipation. Pharmacoeconomics. 2005;23(5):461-76.
3. De Giorgio, R., Ruggeri, E., Stanghellini, V. et al. Chronic constipation in the elderly: a primer for the gastroenterologist. BMC Gastroenterol. 2015;15, 130.
4. Bharucha AE, Lacy BE. Mechanisms, Evaluation, and Management of Chronic Constipation. Gastroenterology. 2020;158(5):1232-1249.e3.
5. Ray G. Evaluation of the Symptom of Constipation in Indian Patients. J Clin Diagn Res. 2016;10(4):OC01-OC3.
6. Miner PB Jr. Elobixibat, the first-in-class Ileal Bile Acid Transporter inhibitor, for the treatment of Chronic Idiopathic Constipation. Expert Opin Pharmacother. 2018 Aug;19(12):1381-1388.
7. Simren M, Abrahamsson H, Bajor A, et al. The IBAT inhibitor A3309—a promising treatment option for patients with chronic idiopathic constipation. Gastroenterology. 2010;5 (Suppl 1:223.
8. Chey WD, Camilleri M, Chang L, Rikner L, Graffner H. A randomized placebo-controlled phase IIb trial of a3309, a bile acid transporter inhibitor, for chronic idiopathic constipation [published correction appears in Am J Gastroenterol. 2014 May;109(5):782]. Am J Gastroenterol. 2011;106(10):1803-1812.
9.
10. Nakajima A, Seki M, Taniguchi S, Ohta A, Gillberg PG, Mattsson JP, Camilleri M. Safety and efficacy of elobixibat for chronic constipation: results from a randomised, double-blind, placebo-controlled, phase 3 trial and an open-label, single-arm, phase 3 trial. Lancet Gastroenterol Hepatol. 2018 Aug;3(8):537-547.
11. O'Donnell LJ, Virjee J, Heaton KW. Detection of pseudodiarrhoea by simple clinical assessment of intestinal transit rate. BMJ. 1990; 300(6722):439-440.

18. APPENDICES

The following documents should be appended with the protocol:

1. Declaration of Helsinki (Current version)
2. List of Study Personnel
3. The Bristol Stool Form Scale (BSFS)[11]

18.1. Appendix 1: Declaration of Helsinki

WORLD MEDICAL ASSOCIATION DECLARATION OF HELSINKI

Ethical Principles for Medical Research Involving Human Subjects

Adopted by the 18th WMA General Assembly, Helsinki, Finland, June 1964

and amended by the:

29th WMA General Assembly, Tokyo, Japan, October 1975

35th WMA General Assembly, Venice, Italy, October 1983

41st WMA General Assembly, Hong Kong, September 1989

48th WMA General Assembly, Somerset West, Republic of South Africa, October 1996

52nd WMA General Assembly, Edinburgh, Scotland, October 2000

53rd WMA General Assembly, Washington DC, USA, October 2002 (Note of Clarification added)

55th WMA General Assembly, Tokyo, Japan, October 2004 (Note of Clarification added)

59th WMA General Assembly, Seoul, Republic of Korea, October 2008

64th WMA General Assembly, Fortaleza, Brazil, October 2013

Preamble

1. The World Medical Association (WMA) has developed the Declaration of Helsinki as a statement of ethical principles for medical research involving human subjects, including research on identifiable human material and data.

The Declaration is intended to be read as a whole and each of its constituent paragraphs should be applied with consideration of all other relevant paragraphs.

2. Consistent with the mandate of the WMA, the Declaration is addressed primarily to physicians. The WMA encourages others who are involved in medical research involving human subjects to adopt these principles.

General Principles

3. The Declaration of Geneva of the WMA binds the physician with the words, “The health of my patient will be my first consideration,” and the International Code of Medical Ethics declares that, “A physician shall act in the patient's best interest when providing medical care.”

4. It is the duty of the physician to promote and safeguard the health, well-being and rights of patients, including those who are involved in medical research. The physician's knowledge and conscience are dedicated to the fulfilment of this duty.

5. Medical progress is based on research that ultimately must include studies involving human subjects.

6. The primary purpose of medical research involving human subjects is to understand the causes, development and effects of diseases and improve preventive, diagnostic and therapeutic interventions (methods, procedures and treatments). Even the best proven interventions must be evaluated continually through research for their safety, effectiveness, efficiency, accessibility and quality.

7. Medical research is subject to ethical standards that promote and ensure respect for all human subjects and protect their health and rights.

8. While the primary purpose of medical research is to generate new knowledge, this goal can never take precedence over the rights and interests of individual research subjects.

9. It is the duty of physicians who are involved in medical research to protect the life, health, dignity, integrity, right to self-determination, privacy, and confidentiality of personal information of research subjects. The responsibility for the protection of research subjects must always rest with the physician or other health care professionals and never with the research subjects, even though they have given consent.

10. Physicians must consider the ethical, legal and regulatory norms and standards for research involving human subjects in their own countries as well as applicable international norms and standards. No national or international ethical, legal or regulatory requirement should reduce or eliminate any of the protections for research subjects set forth in this Declaration.

11. Medical research should be conducted in a manner that minimises possible harm to the environment.

12. Medical research involving human subjects must be conducted only by individuals with the appropriate ethics and scientific education, training and qualifications. Research on patients or healthy volunteers requires the supervision of a competent and appropriately qualified physician or other health care professional.

13. Groups that are underrepresented in medical research should be provided appropriate access to participation in research.

14. Physicians who combine medical research with medical care should involve their patients in research only to the extent that this is justified by its potential preventive, diagnostic or therapeutic value and if the physician has good reason to believe that participation in the research study will not adversely affect the health of the patients who serve as research subjects.

15. Appropriate compensation and treatment for subjects who are harmed as a result of participating in research must be ensured.

Risks, Burdens and Benefits

16. In medical practice and in medical research, most interventions involve risks and burdens.

Medical research involving human subjects may only be conducted if the importance of the objective outweighs the risks and burdens to the research subjects.

17. All medical research involving human subjects must be preceded by careful assessment of predictable risks and burdens to the individuals and groups involved in the research in comparison with foreseeable benefits to them and to other individuals or groups affected by the condition under investigation.

Measures to minimise the risks must be implemented. The risks must be continuously monitored, assessed and documented by the researcher.

18. Physicians may not be involved in a research study involving human subjects unless they are confident that the risks have been adequately assessed and can be satisfactorily managed.

When the risks are found to outweigh the potential benefits or when there is conclusive proof of definitive outcomes, physicians must assess whether to continue, modify or immediately stop the study.

Vulnerable Groups and Individuals

19. Some groups and individuals are particularly vulnerable and may have an increased likelihood of being wronged or of incurring additional harm.

All vulnerable groups and individuals should receive specifically considered protection.

20. Medical research with a vulnerable group is only justified if the research is responsive to the health needs or priorities of this group and the research cannot be carried out in a non-vulnerable group. In addition, this group should stand to benefit from the knowledge, practices or interventions that result from the research.

Scientific Requirements and Research Protocols

21. Medical research involving human subjects must conform to generally accepted scientific principles, be based on a thorough knowledge of the scientific literature, other relevant sources of information, and adequate laboratory and, as appropriate, animal experimentation. The welfare of animals used for research must be respected.

22. The design and performance of each research study involving human subjects must be clearly described and justified in a research protocol.

The protocol should contain a statement of the ethical considerations involved and should indicate how the principles in this Declaration have been addressed. The protocol should include information regarding funding, sponsors, institutional affiliations, potential conflicts of interest, incentives for subjects and information regarding provisions for treating and/or compensating subjects who are harmed as a consequence of participation in the research study.

In clinical trials, the protocol must also describe appropriate arrangements for post-trial provisions.

Research Ethics Committees

23. The research protocol must be submitted for consideration, comment, guidance and approval to the concerned research ethics committee before the study begins. This committee must be transparent in its functioning, must be independent of the researcher, the sponsor and any other undue influence and must be duly qualified. It must take into consideration the laws and regulations of the country or countries in which the research is to be performed as well as applicable international norms and standards but these must not be allowed to reduce or eliminate any of the protections for research subjects set forth in this Declaration.

The committee must have the right to monitor ongoing studies. The researcher must provide monitoring information to the committee, especially information about any serious adverse events. No amendment to the protocol may be made without consideration and approval by the committee. After the end of the study, the researchers must submit a final report to the committee containing a summary of the study's findings and conclusions.

Privacy and Confidentiality

24. Every precaution must be taken to protect the privacy of research subjects and the confidentiality of their personal information.

Informed Consent

25. Participation by individuals capable of giving informed consent as subjects in medical research must be voluntary. Although it may be appropriate to consult family members or community leaders, no individual capable of giving informed consent may be enrolled in a research study unless he or she freely agrees.

26. In medical research involving human subjects capable of giving informed consent, each potential subject must be adequately informed of the aims, methods, sources of funding, any possible conflicts of interest, institutional affiliations of the researcher, the anticipated benefits and potential risks of the study and the discomfort it may entail, post-study provisions and any other relevant aspects of the study. The potential subject must be informed of the right to refuse to participate in the study or to withdraw consent to participate at any time without reprisal. Special attention should be

given to the specific information needs of individual potential subjects as well as to the methods used to deliver the information.

After ensuring that the potential subject has understood the information, the physician or another appropriately qualified individual must then seek the potential subject's freely-given informed consent, preferably in writing. If the consent cannot be expressed in writing, the non-written consent must be formally documented and witnessed.

All medical research subjects should be given the option of being informed about the general outcome and results of the study.

27. When seeking informed consent for participation in a research study the physician must be particularly cautious if the potential subject is in a dependent relationship with the physician or may consent under duress. In such situations the informed consent must be sought by an appropriately qualified individual who is completely independent of this relationship.

28. For a potential research subject who is incapable of giving informed consent, the physician must seek informed consent from the legally authorised representative. These individuals must not be included in a research study that has no likelihood of benefit for them unless it is intended to promote the health of the group represented by the potential subject, the research cannot instead be performed with persons capable of providing informed consent, and the research entails only minimal risk and minimal burden.

29. When a potential research subject who is deemed incapable of giving informed consent is able to give assent to decisions about participation in research, the physician must seek that assent in addition to the consent of the legally authorised representative. The potential subject's dissent should be respected.

30. Research involving subjects who are physically or mentally incapable of giving consent, for example, unconscious patients, may be done only if the physical or mental condition that prevents giving informed consent is a necessary characteristic of the research group. In such circumstances the physician must seek informed consent from the legally authorised representative. If no such representative is available and if the research cannot be delayed, the study may proceed without informed consent provided that the specific reasons for involving subjects with a condition that renders them unable

to give informed consent have been stated in the research protocol and the study has been approved by a research ethics committee. Consent to remain in the research must be obtained as soon as possible from the subject or a legally authorised representative.

31. The physician must fully inform the patient which aspects of their care are related to the research. The refusal of a patient to participate in a study or the patient's decision to withdraw from the study must never adversely affect the patient-physician relationship.

32. For medical research using identifiable human material or data, such as research on material or data contained in biobanks or similar repositories, physicians must seek informed consent for its collection, storage and/or reuse. There may be exceptional situations where consent would be impossible or impracticable to obtain for such research. In such situations the research may be done only after consideration and approval of a research ethics committee.

Use of Placebo

33. The benefits, risks, burdens and effectiveness of a new intervention must be tested against those of the best proven intervention(s), except in the following circumstances:

Where no proven intervention exists, the use of placebo, or no intervention, is acceptable; or Where for compelling and scientifically sound methodological reasons the use of any intervention less effective than the best proven one, the use of placebo, or no intervention is necessary to determine the efficacy or safety of an intervention and the patients who receive any intervention less effective than the best proven one, placebo, or no intervention will not be subject to additional risks of serious or irreversible harm as a result of not receiving the best proven intervention.

Extreme care must be taken to avoid abuse of this option.

Post-Trial Provisions

34. In advance of a clinical trial, sponsors, researchers and host country governments should make provisions for post-trial access for all participants who still need an intervention identified as beneficial in the trial. This information must also be disclosed to participants during the informed consent process.

Research Registration and Publication and Dissemination of Results

35. Every research study involving human subjects must be registered in a publicly accessible database before recruitment of the first subject.

36. Researchers, authors, sponsors, editors and publishers all have ethical obligations with regard to the publication and dissemination of the results of research. Researchers have a duty to make publicly available the results of their research on human subjects and are accountable for the completeness and accuracy of their reports. All parties should adhere to accepted guidelines for ethical reporting. Negative and inconclusive as well as positive results must be published or otherwise made publicly available. Sources of funding, institutional affiliations and conflicts of interest must be declared in the publication. Reports of research not in accordance with the principles of this Declaration should not be accepted for publication.

Unproven Interventions in Clinical Practice

37. In the treatment of an individual patient, where proven interventions do not exist or other known interventions have been ineffective, the physician, after seeking expert advice, with informed consent from the patient or a legally authorized representative, may use an unproven intervention if in the physician's judgment it offers hope of saving life, re-establishing health or alleviating suffering. This intervention should subsequently be made the object of research, designed to evaluate its safety and efficacy. In all cases, new information must be recorded and, where appropriate, made publicly available.

18.2. Appendix 2: List of Study Personnel

**Sponsor**	Dr. Reddy's Laboratories Ltd.<br>Integrated Product Development,<br>Innovation plaza,<br>Survey No: 42, 45 and 46,<br>Bachupally village, Bachupally Mandal<br>Medchal Malkajgiri District,<br>Hyderabad - 500 090,<br>Telangana, India.
**Sponsor Contact**	Dr. Piyush Agarwal<br>Head Clinical Development & Clinical Strategy<br>Dr. Reddy's Laboratories Ltd.<br>Email ID: piyushagarwal@drreddys.com
**Medical Monitor**	Dr. Dhruv Mahendru, M.D<br>Medical Monitor<br>Email ID: dhruvmahendru@drreddys.com<br>OR<br>Dr. Sanjana Dawra, M.D<br>Medical Monitor<br>Email ID: sanjanad@drreddys.com
**Project Manager**	Vipul Modh.<br>Clinical Research Specialist, –Clinical & Medical Operations<br>Integrated Product Development Organization, Innovation plaza,<br>Survey No: 42, 45 and 46,<br>Bachupally village, Bachupally Mandal Medchal-Malkajgiri District - 500 090, Telangana, India.<br>Email: vipulmodh@drreddys.com<br>Tel: +91 40 4879 6017
**Study Monitors**	From –<br>Global Clinical Management<br>Dr. Reddy's Laboratories Ltd.<br>Integrated Product Development,<br>Innovation plaza,<br>Survey No: 42, 45 and 46,<br>Bachupally village, Bachupally Mandal<br>Medchal-Malkajgiri District,<br>Hyderabad - 500 090,
**Pharmacovigilance Team (Safety/ Serious Adverse Event Reporting)**	Email ID: pharmacovigilance@drreddys.com<br>nitus@drreddys.com<br>*Tel: +91 40 4900 2429*

18.3. Appendix 3. The Bristol Stool Form Scale (BSFS)

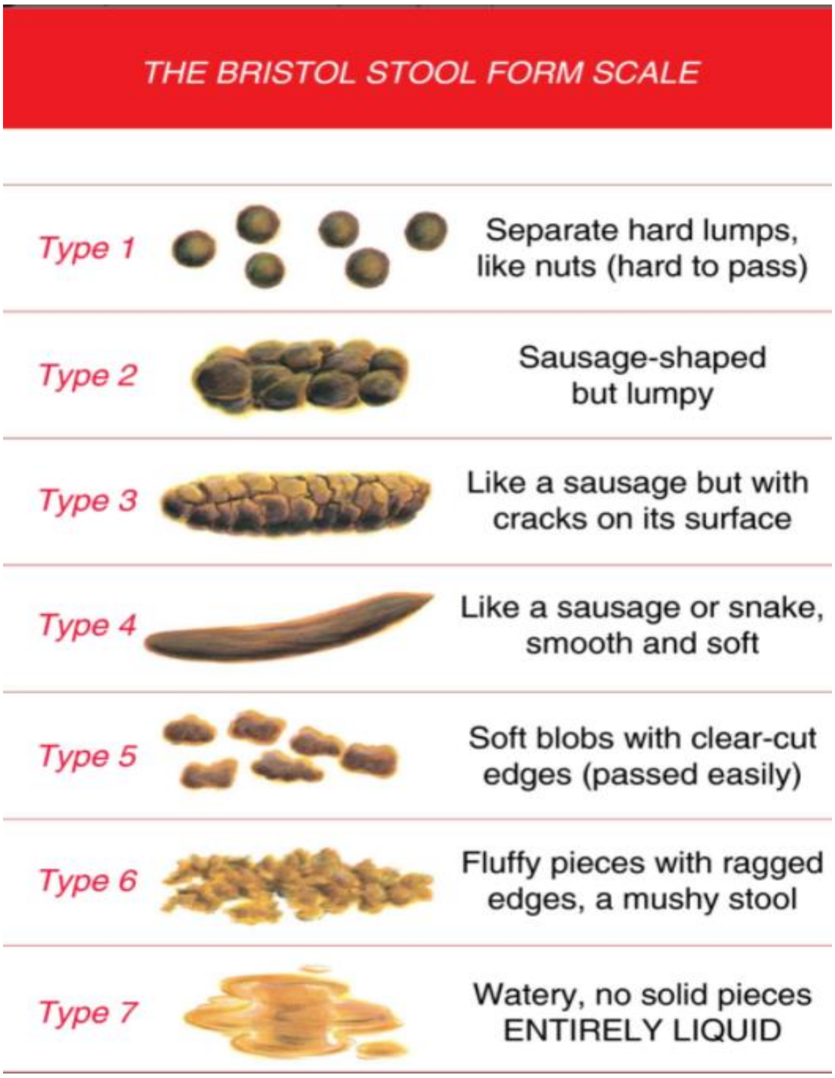

Adapted from: O'Donnell LJ, Virjee J, Heaton KW. Detection of pseudodiarrhoea by simple clinical assessment of intestinal transit rate. BMJ. 1990; 300(6722):439-440.
