# Supplementary material for: Efficacy and safety of elobixibat in patients with chronic constipation—A randomized, multicenter, double-blind, placebo-controlled, parallel-group study from India
Source: Indian J Gastroenterol. 2025 Feb 22;44(3):336–44. doi: 10.1007/s12664-024-01719-7 (PMC12141165; doi:10.1007/s12664-024-01719-7)
Supplement: Supplementary file 3 — Supplementary file3 (DOCX 16 KB) [file 12664_2024_1719_MOESM3_ESM.docx]

Supplementary File: List of Investigators

| **Site No.** | **Name of the Investigator** | **Address** |
| --- | --- | --- |
| 01 | Dr. Kabrawala Mayank Vasantlal | SIDS Hospital & Research centre, A unit of SIDS health care private limited, Off ring road, Near shell petrol pump,  Ring road-sosyo circle lane, Surat-395002, Gujarat, India. |
| 02 | Dr. Vinay Kumar | GSVM Medical College, Post Graduate Department of Medicine, GSVM Medical College, Kanpur - 208002, Uttar Pradesh |
| 03 | Dr. Omesh Goyal | Dayanand Medical College and Hospital, Civil lines, Tagore Nagar, Ludhiana, Punjab – 141001 |
| 04 | Dr. Mukesh Kalla | S.R. Kalla Memorial Gastro and General Hospital,  78-79 Dhuleshwar Garden, Behind HSBC Bank, Sardar Patel Marg, C-Scheme, Jaipur - 302001, Rajasthan |
| 05 | Dr. Mukewar Shrikant Vasantrao | Midas Multispeciality Hospital Pvt. Ltd. Midas Heights, 07 Central Bazzar Road, Ramdaspeth, Nagpur – 440010, Maharashtra |
| 06 | Dr. Ambanna Gowda | Citizen Hospital, #14, 2nd Main, Dispensary Road, Kalasipalya, Bangalore – 560002, Karnataka |
| 07 | Dr. B Ravi Shankar | Yashoda Hospitals, Behind Hari Hara Kala Bhavan, SP Road, Secunderabad - 500003, Telengana |
| 08 | Dr. KN Chandan Kumar | Gleneagles Global Hospitals, 6-1-1070/ 1 to 4, Lakdi-ka-pool, Hyderabad - 500004, Telangana, |
| 09 | Dr. Manas Kumar Panigrahi | AIIMS, Bhubaneshwar, Sijua, Patrapada, Dumduma, Odisha, India - 751019 |
| 10 | Dr. Anumula Kavitha | Government General Hospital, Department of Gastroenterology,  Room No. 220, 2^nd^ Floor, Jubilee Block, Guntur-522001, Andhra Pradesh |
| 11 | Dr. Abhijit Chandra | Department of Surgical Gastroenterology, Gandhi Memorial and associated Hospitals, King George’s Medical University, (U.P), Lucknow-226003 (U.P) |
| 12 | Dr. Ravindra Gaadhe | Gastroplus Digestive Disease Centre, D-Block, 3^rd^ Floor, Galaxy Bazaar, Sunrise Park Road, Vastrapur, Ahmedabad-380054, Gujarat |
| 13 | Dr. B. Sivananda Reddy | Aware Gleneagles Global Hospitals,  08-16-01, Near Sagar X Road, Saroor Nagar, L B Nagar,  Hyderabad – 500 035, Telangana. |
| 14 | Dr. Parmar Kartikeya Ganpatlal | Department of Medicine, B. J. Medical College & Civil Hospital, Asarwa, Ahmedabad -380016, Gujarat, India |
| 15 | Dr. Ujwal Gajula | Excel Hospital, # 1-5-56/29, Old Alwal, Near IG Statue, Alwal, Secunderabad – 500010, Telangana, India |
| 16 | Dr. Vipul Khandelwal | Apex Hospitals Pvt. Ltd, S P 4 & 6, Malviya Industrial Area,  Malviya Nagar, Jaipur, Rajasthan – 302017, India. |
